# Training–Fuel Coupling (TFC): A Molecular Sports Nutrition Framework for Energy Availability, Chrono-Nutrition, and Performance Optimization

**DOI:** 10.3390/nu18040693

**Published:** 2026-02-21

**Authors:** Mirela Stoian, Dan Cristian Mănescu

**Affiliations:** 1 Faculty of AgriFood and Environmental Economics, Bucharest University of Economic Studies, 010374 Bucharest, Romania; mirela.stoian@eam.ase.ro; 2 Department of Physical Education and Sports, Faculty of AgriFood and Environmental Economics, Bucharest University of Economic Studies, 010374 Bucharest, Romania

**Keywords:** sports nutrition, molecular nutrition, energy availability, chrono-nutrition, AMPK–mTOR signaling, nutritional periodization, performance optimization, RED-S

## Abstract

In sports nutrition, performance adaptation emerges from the coordinated molecular interaction between physical training and nutrient availability. This narrative review with conceptual synthesis advances Training–Fuel Coupling (TFC) as a systems physiology framework that conceptualizes nutrient availability, timing, and recovery feeding as molecular control variables proposed to govern exercise-induced adaptation. Integrating evidence from exercise metabolism and nutritional science, the model conceptualizes how substrate availability may modulate the dynamic crosstalk between AMP-activated protein kinase (AMPK) and mechanistic target of rapamycin (mTOR), shaping metabolic flexibility, anabolic recovery, and long-term performance optimization. Low-energy and low-glycogen contexts preferentially activate AMPK-dependent pathways supporting mitochondrial remodeling and oxidative efficiency, whereas nutrient-replete states facilitate mTOR-mediated protein synthesis and structural restoration. When strategically alternated through chrono-nutrition and nutritional periodization, these energetic states are hypothesized to generate oscillatory signaling patterns that enhance adaptive efficiency while limiting chronic metabolic strain. From a sports nutrition perspective, TFC provides a mechanistic rationale for energy availability management, recovery nutrition, and the prevention of maladaptive states such as Relative Energy Deficiency in Sport (RED-S). By reframing nutrients as regulatory signals rather than passive fuel, this framework integrates molecular nutrition with performance physiology, offering a unifying, systems-level and hypothesis-generating perspective on training–nutrition interactions that delineates testable pathways for future empirical investigation.

## 1. Introduction

Optimizing human performance requires more than balancing training load and energy intake—it requires understanding how energy metabolism and molecular signaling co-regulate adaptation. Traditional models of exercise metabolism frame nutrients as fuel and training as stimulus. Yet, mounting evidence shows that nutrients also serve as signals shaping molecular pathways and long-term adaptations [1,2,3,4,5].

Within sports nutrition, this perspective has shifted attention from macronutrient quantity toward energy availability, nutrient timing, and context-dependent signaling as key determinants of exercise-induced adaptation. Nutrients are increasingly recognized as regulatory inputs that interact with cellular energy-sensing networks, modulating the balance between catabolic and anabolic pathways. Through this lens, variations in substrate availability are proposed to shape how training stress is processed at the molecular level, influencing mitochondrial remodeling, protein synthesis, and recovery efficiency across repeated training cycles [6,7,8,9,10].

The Training–Fuel Coupling (TFC) concept captures this bidirectional relationship: training alters the metabolic environment, and the metabolic environment influences how training is interpreted by the cell. This coupling links the energetic state of the muscle to adaptive pathways like mitochondrial biogenesis, hypertrophy, or fatigue resistance through the dynamic interplay of AMP-activated protein kinase (AMPK) and the mechanistic target of rapamycin (mTOR) signaling.

Despite abundant empirical data, there is still no unifying systems-level framework that explains when and how nutrient timing and availability modulate performance adaptations. This study therefore proposes a mechanistic conceptual model based on systems physiology and control theory principles, treating adaptation of the organism as an emergent property of interacting regulatory components and feedback processes—representing system-level regulation—rather than as the result of isolated molecular pathways. Within this view, the organism is modeled as a self-regulating energetic system with defined inputs (exercise), controllers (signaling networks), and outputs (adaptation, performance). In this context, Training–Fuel Coupling (TFC) is used to denote a systems-level regulatory construct in which training-induced energetic stress and nutrient availability act as coordinated control inputs shaping adaptive signaling, rather than as a single measurable physiological variable.

In line with this objective, the manuscript is structured as a narrative review with conceptual synthesis. Rather than establishing causal effects or advancing a validated regulatory theory, it integrates heterogeneous mechanistic evidence from exercise physiology and sports nutrition into a coherent systems-level framework. Within this scope, Training–Fuel Coupling (TFC) framework is further articulated as a heuristic, hypothesis-generating model, intended to organize existing knowledge, clarify regulatory logic, and derive testable hypotheses regarding the interaction between training load, nutrient availability, and adaptive signaling.

We outline four model-derived hypotheses emerging from the Training–Fuel Coupling framework:

**H1**: *Variability in energetic state across training sessions enhances adaptation efficiency up to a physiological threshold.*

**H2**: *Individual glycogen and amino acid availability thresholds determine the dominance of AMPK- versus mTOR-mediated signaling.*

**H3**: *Periodized manipulation of nutrient timing (e.g., train-low, lift-fed) produces oscillatory signaling patterns that optimize long-term adaptation.*

**H4**: *Continuous metabolic monitoring—such as continuous glucose monitoring (CGM), heart rate variability (HRV), and oxygen kinetics (O_2_ kinetics)—can support closed-loop control of training based on Training–Fuel Coupling (TFC) principles.*

Literature Review

Physiological adaptation to exercise cannot be understood apart from the energetic context in which it occurs. Over the past two decades, the concept of metabolic flexibility has redefined performance as a measurable physiological capacity describing the ability of the organism to adjust substrate utilization and dominant signaling pathways in response to changing energetic demands, rather than as a static notion of energetic efficiency. This capacity to shift efficiently between carbohydrate and lipid oxidation becomes the pivot of any model that attempts to link training-induced stress with nutritional support—including the Training–Fuel Coupling (TFC) model proposed here, in which metabolic flexibility functions as a regulatory control variable determining the efficiency of adaptation [11,12,13,14,15].

Research on glycogen availability has consistently shown that glycogen functions not merely as an energy reserve but as a contextual sensor. Its level directly influences the activation of AMPK and the transcription of peroxisome proliferator-activated receptor gamma coactivator 1-alpha (PGC-1α), and “train-low” strategies intensify oxidative signaling and mitochondrial biogenesis. However, when this strategy becomes chronic, anabolic capacity is reduced. Thus, glycogen appears as a switching threshold between two distinct metabolic regimes—the exact role assigned to it in the TFC hypothesis of adaptive regulation [16,17,18,19,20].

At the center of this balance lies the AMPK–mTOR crosstalk, a genuine logic gate of the system. Molecular literature demonstrates that AMPK activation inhibits mechanistic target of rapamycin complex 1 (mTORC1) when energy is low, while leucine and insulin intake reactivate the anabolic pathway. This alternation between inhibition and activation is what TFC describes as functional oscillation—a periodic succession of catabolic and anabolic states designed to maximize adaptation. Similarly, data on essential amino acid supplementation confirm that anabolism depends on the synchronization between protein intake and energetic status, not on absolute quantity [21,22,23,24,25].

These discoveries have given rise to a new paradigm: nutritional periodization. A series of studies have shown that deliberate variations in carbohydrate and protein intake between sessions train the metabolic system as effectively as variations in volume or intensity. Training–Fuel Coupling offers a theoretical framework for interpreting this logic: the calculated alternation of energetic states maintains the dual sensitivity of AMPK–mTOR, preventing signal desensitization and optimizing adaptive efficiency [26,27,28,29,30].

An important reinterpretation of the literature on concurrent training (endurance and strength) supports the same idea. The so-called “interference effect” does not arise from the overlap of stimuli but from energetic misalignment between the two exercise modalities. TFC provides a heuristic lens suggesting that some reported “interference” effects may be partly explained by energetic and nutritional timing—i.e., how concurrent sessions are positioned relative to glycogen availability and anabolic sensitivity—rather than by an unavoidable incompatibility between modalities [31,32,33,34,35].

Likewise, evidence concerning chronobiology of metabolism suggests that the time of day and the post-exercise window modify nutrient sensitivity and gene expression responses. Circadian oscillations of AMPK and mTOR activity confirm that time acts as a dimension of regulation, not a passive context. Within this view, TFC integrates the temporal factor as part of the system’s control, proposing a rhythmic physiology of adaptation [36,37,38,39,40].

As research on redox signaling and the Sirtuin-1 NAD^+^-dependent deacetylase (SIRT1/NAD^+^) axis has advanced, where sirtuin-1 (SIRT1) activity depends on nicotinamide adenine dinucleotide (NAD^+^) availability, it has become evident that moderate oxidative stress is also a component of adaptive regulation. Reactive oxygen species are not merely by-products but can act as calibrating signals capable of modulating the amplitude of AMPK and PGC-1α responses. In the TFC model, this redox loop operates as a fine feedback mechanism, adjusting the “volume” of the signal without altering its logical structure [41,42,43,44,45].

Recent literature also emphasizes that nutritional recovery is not a pause but the anabolic phase of the same adaptive cycle. Glycogen repletion and protein synthesis do not merely restore stores but reset thresholds for the next session. Thus, recovery becomes an active input of the system, not a passive interval between stimuli—a central principle in TFC [46,47,48,49,50].

Individual differences, often cited as evidence of adaptive unpredictability, can be understood in terms of mobile thresholds. “Non-responders” are not biological exceptions but individuals whose energetic oscillation fails to reach the optimal regulatory frequency. In this interpretation, personalization of training becomes a matter of calibrating the energetic cycle rather than rewriting mechanisms [51,52,53,54,55].

Technological advances have enabled continuous monitoring of metabolic signals—glucose, HRV, lactate, and peripheral temperature—offering insight into the system’s energetic position. Practically, these variables become the “observables” of the control loop proposed by TFC, allowing real-time regulation of metabolic oscillations [56,57,58,59,60].

Viewed in isolation, many experimental findings appear contradictory. Some studies show benefits of carbohydrate restriction, others of supercompensation; some emphasize intensity, others frequency. TFC offers an integrative interpretation in which some apparently divergent findings may correspond to different energetic phases or experimental contexts within a broader oscillatory regulation concept [61,62,63,64]. Rather than rejecting paradoxes, it integrates them into a shared logic of adaptive regulation [61,62,63,64].

Ultimately, converging evidence on AMPK–mTOR crosstalk, “train-low/recover-high” strategies, and endogenous energetic rhythms supports the idea that optimal performance results from an intelligent alternation of energetic states, not stability. Training–Fuel Coupling aims to organize these dispersed observations into a coherent, testable architecture of physiological control, where stress and recovery emerge as complementary phases of the same adaptive equation [65,66,67,68,69].

## 2. Methodological Approach

Given its narrative and conceptual nature, this study does not report original experimental data. Instead, it adopts a narrative review design c ombined with conceptual synthesis, aiming to integrate mechanistic evidence from multiple domains and to derive system-level hypotheses rather than to perform quantitative effect estimation.

### 2.1. Narrative Review Design and Conceptual Synthesis

This study was conducted as a narrative review with conceptual synthesis, designed to integrate mechanistic evidence from exercise physiology, nutrition science, and systems biology into a unified theoretical framework termed Training–Fuel Coupling (TFC). The methodological objective was not quantitative effect estimation, but the identification, organization, and integration of mechanistic patterns that explain how energetic context modulates adaptive signaling and performance outcomes.

Given the complexity of AMPK–mTOR crosstalk, substrate availability thresholds, and temporal regulation of adaptation, a narrative review approach was selected as the most appropriate methodological design. This format allows the synthesis of heterogeneous evidence—including human intervention studies, molecular experiments, animal models, and theoretical physiology—into a coherent explanatory architecture that cannot be captured by meta-analytic aggregation alone.

Literature Search Strategy—a structured but non-exhaustive literature search was conducted to identify studies relevant to the interaction between training load, nutrient availability, and molecular signaling pathways governing adaptation. The primary databases consulted included PubMed, Scopus, and Web of Science, covering publications from 2000 to 2025, a period corresponding to the maturation of molecular exercise physiology and nutritional periodization research.

Search terms were combined using Boolean logic and included keywords related to: exercise metabolism, energy availability, glycogen, AMPK, mTOR, PGC-1α, SIRT1, nutritional periodization, train-low strategies, concurrent training, metabolic flexibility, and systems physiology. Reference lists of key reviews and landmark articles were also screened to identify additional conceptually relevant sources.

The search strategy prioritized mechanistic relevance and theoretical contribution over completeness. As such, studies were selected based on their ability to elucidate regulatory relationships, signaling thresholds, temporal dynamics, or feedback mechanisms linking energetic state to adaptive outcomes.

Study Selection and Inclusion Criteria—included studies met one or more of the following criteria:

1. Experimental or theoretical work describing molecular regulators of energy sensing and growth signaling (e.g., AMPK, mTORC1, SIRT1, PGC-1α).
2. Human or animal studies examining the effects of substrate availability, glycogen manipulation, or nutrient timing on training adaptation.
3. Research addressing nutritional or training periodization, including “train-low”, “sleep-low”, fasted vs. fed training, and concurrent training paradigms.
4. Conceptual or systems-level papers providing theoretical frameworks for metabolic regulation, feedback control, or adaptive dynamics.

No formal exclusion criteria based on sample size, population, or study design were imposed, as the aim was conceptual integration rather than statistical inference. Review articles were included when they contributed theoretical clarity or summarized mechanistic consensus.

Conceptual Synthesis and Systems Integration—rather than pooling outcomes quantitatively, the selected literature was reorganized through a systems physiology lens, treating the organism as a self-regulating energetic system. Evidence was mapped across three interacting layers:

5. Control inputs—training load, intensity, and nutrient availability.
6. Regulatory controllers—molecular signaling networks centered on AMPK, mTORC1, and SIRT1.
7. Adaptive outputs—mitochondrial remodeling, protein synthesis, performance capacity, and recovery efficiency.

This mapping enabled the identification of recurrent regulatory motifs—reciprocal inhibition, threshold switching, oscillatory dominance, and feedback adjustment—which were subsequently formalized into the Training–Fuel Coupling framework. Hypotheses (H1–H4) were derived through analytical deduction based on the internal logic of this integrated system rather than post hoc interpretation of isolated findings.

Analytical Deduction and Model Validation—the conceptual model was evaluated through internal logical validation and external correspondence with empirical literature. Internal validation ensured consistency with established biological principles, including energy sensing polarity, signaling hierarchy, and feedback directionality. External validation assessed whether the predicted behaviors of the model—energetic variability, threshold regulation, oscillatory coupling, and closed-loop adaptation—were congruent with observed training and nutritional phenomena reported in experimental studies.

This dual validation approach aligns with best practices for theory-building narrative reviews, where robustness is achieved through coherence, explanatory power, and empirical plausibility rather than statistical aggregation.

Scope and Methodological Boundaries—this narrative review focuses primarily on skeletal muscle metabolism and performance adaptation, acknowledging that other regulatory systems (endocrine, immune, behavioral) are discussed only insofar as they interact with energetic control. Molecular thresholds and composite indices proposed within the TFC framework represent context-dependent estimates, intended to guide hypothesis generation rather than define universal physiological constants.

As a theory-building narrative review, this synthesis is inherently subject to selection bias, insofar as the included literature was prioritized for mechanistic relevance and conceptual contribution rather than exhaustive coverage. To mitigate this risk, evidence was drawn from multiple experimental levels (human, animal, and molecular), and contradictory or context-dependent findings were explicitly considered and integrated within the proposed systems framework rather than excluded.

Throughout the manuscript, causal language is used to denote conceptual, model-internal relationships, consistent with the hypothesis-generating scope of this narrative review.

Accordingly, the Training–Fuel Coupling model should be interpreted as a conceptual, hypothesis-generating framework whose validity rests on explanatory coherence and empirical plausibility, providing a structured basis for future experimental testing, longitudinal monitoring, and individualized training–nutrition optimization.

Relation to Existing Conceptual Frameworks—the Training–Fuel Coupling (TFC) framework was developed with explicit reference to existing conceptual models addressing training–nutrition interactions, including the glycogen threshold hypothesis, nutritional periodization approaches, metabolic flexibility frameworks, and models of concurrent training adaptation.

The glycogen threshold hypothesis provides a mechanistic account of AMPK-dominant signaling under low-carbohydrate conditions but primarily addresses acute molecular responses and does not formalize recovery-driven anabolic reactivation or longer-term regulatory dynamics. Nutritional periodization models extend this logic into applied practice but remain largely descriptive, without explicitly defining the regulatory structure linking energetic state, signaling dominance, and adaptive outcomes.

Metabolic flexibility frameworks conceptualize substrate switching capacity as a physiological trait, typically operationalized through oxidation measures, yet do not explicitly model signaling thresholds, oscillatory dominance, or feedback regulation across training cycles. Similarly, concurrent training models often describe interference phenomena without formalizing energetic context as a governing variable.

The TFC framework does not seek to replace these models. Rather, it integrates their core insights into a systems-level, hypothesis-generating construct that emphasizes energetic variability, threshold-dependent switching, oscillatory coupling, and closed-loop adaptation. This positioning defines TFC as an interpretative and organizational framework, not as an alternative mechanistic theory.

### 2.2. Conceptual Model Development

The development of the Training–Fuel Coupling (TFC) framework followed an analytical path grounded in systems physiology. Mechanistic evidence from exercise and nutrition research was reorganized within a unifying model that captures the regulatory interplay between energetic stress, molecular signaling, and adaptive outcomes. This methodological architecture was designed to formalize the logic through which training and substrate availability jointly determine the efficiency of physiological adaptation [70,71,72].

This model-building process was designed as a conceptual systems analysis aimed at developing a mechanistic framework linking training stimulus, substrate availability, and adaptive performance outcomes. Rather than collecting new experimental data, the research follows a model-building approach—integrating established physiological and molecular findings into a unified regulatory architecture termed the Training–Fuel Coupling (TFC) model [73].

The conceptual model development consisted of four sequential phases:

8. Evidence Mapping: identification and organization of mechanistic studies describing interactions among exercise, nutrition, and molecular signaling (AMPK, mTOR, SIRT1).
9. Systems Integration: synthesis of these mechanisms into a conceptual control model connecting energetic inputs, signaling controllers, and adaptive outputs.
10. Analytical Deduction: derivation of system-level hypotheses describing expected behaviors under variable energetic and nutritional conditions.
11. Model Validation: internal verification of logical consistency through diagrammatic reasoning and external consistency through correspondence with empirical literature.

**Figure 1:**
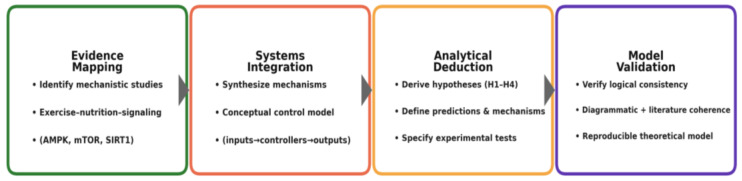
Schematic of the model-building workflow used in this study, showing the left-to-right progression from evidence aggregation to internal validation. Chevrons indicate directional dependence between phases; colors distinguish phase roles without implying quantitative weight.

This structure mirrors the logic of experimental design, where data are replaced by mechanistic evidence and statistical testing by systemic coherence analysis. The resulting framework constitutes a reproducible theoretical model capable of generating testable predictions for future empirical studies [21,74,75,76,77,78,79] (Figure 1).

The diagram functions as a methodological scaffold rather than a result: it formalizes how mechanistic findings are consolidated into a control architecture, from which system-level hypotheses (H1–H4) are analytically derived and then internally checked for coherence with established physiology. By separating evidence mapping, integrative modeling, hypothesis deduction, and internal validation, the workflow preserves inferential transparency, minimizes circularity, and yields a reproducible theoretical product that generates empirically testable predictions for subsequent studies.

### 2.3. Evidence Mapping

The first analytical phase consisted of mapping the existing mechanistic evidence describing how energetic stress, nutrient availability, and molecular signaling interact to determine training adaptation. Rather than a bibliometric survey, this phase functioned as a conceptual synthesis, extracting the structural logic of published findings in exercise metabolism and nutritional periodization. Studies were selected according to their contribution to mechanistic understanding—those that experimentally or theoretically linked training stimulus, substrate flux, and molecular regulators such as AMPK, mTOR, and SIRT1 [80,81].

The mapping process identified three complementary domains [82]. The first concerned the molecular controllers of energy and growth—AMPK as the principal energy sensor activated by low glycogen and ATP depletion, and mTORC1 as the key anabolic switch responsive to amino acid and insulin signaling. The second domain involved nutritional modulation, summarizing how carbohydrate and protein availability alter signaling kinetics across recovery and adaptation cycles. The third domain addressed training configuration, integrating endurance, resistance, and concurrent protocols that manipulate energy turnover and substrate oxidation. Collectively, these domains define the functional space within which metabolic regulation occurs [83].

From this mapping, patterns emerged that transcend individual studies. Repeatedly, evidence converged on the idea that alternating between energetic scarcity and abundance enhances adaptive signaling plasticity. Endurance trials under low-glycogen conditions amplified AMPK activity and mitochondrial biogenesis, whereas subsequent nutrient-rich recovery restored mTOR activation and protein synthesis. Resistance training combined with amino acid availability reinforced similar alternation effects, emphasizing temporal coupling between metabolic state and adaptive outcome. These consistencies provided the empirical foundation for treating energy state as a control variable rather than a background condition.

The resulting evidence map was organized as a matrix linking energetic context, dominant signaling pathway, and adaptive phenotype. Low-energy states aligned with oxidative remodeling and stress resilience; high-energy states with anabolic reconstruction and tissue growth. Transitional zones—moderate glycogen availability, mixed macronutrient recovery—produced hybrid adaptations characterized by efficiency gains without maximal hypertrophy. This structure clarified how substrate availability and training modality interact as dual inputs governing adaptation efficiency.

By systematizing dispersed findings into this logic matrix, the mapping phase established the empirical boundaries of the Training–Fuel Coupling framework [81,84]. It delineated where mechanistic consensus exists, where contradictions arise, and where theoretical modeling can extend interpretation. The process therefore converted a heterogeneous literature into a structured conceptual terrain from which the next phase, Systems Integration, could formalize the underlying control architecture.

### 2.4. Systems Integration

The integration phase translated the mapped evidence into a structured control model representing how training and nutritional inputs interact to regulate adaptive signaling. Building upon the relationships identified in the mapping stage, the Training–Fuel Coupling (TFC) framework was organized according to the logic of systems physiology: adaptive outcomes arise from the coordination of regulatory loops that maintain energy balance while promoting functional remodeling [81].

The model defines two primary control inputs. The first is training intensity and volume, which determine the magnitude of energetic stress through ATP turnover, glycogen depletion, and metabolite accumulation. The second is nutrient availability, modulating the degree of metabolic recovery and anabolic signaling through glucose, amino acids, and insulin responses. These inputs feed into three interacting regulatory controllers—AMPK, mTORC1, and SIRT1—each acting as a node in a coupled feedback network. AMPK functions as a negative feedback sensor, activated under energy deficit to restore balance through oxidative metabolism and substrate mobilization. mTORC1 serves as a positive feedback amplifier, driving protein synthesis and cellular growth under nutrient-rich conditions. SIRT1 acts as a modulator linking energy state to transcriptional control, fine-tuning both catabolic and anabolic responses [85,86].

The coupling of these controllers can be conceptualized as a bi-directional regulatory loop: exercise-induced energy stress activates AMPK and suppresses mTORC1, initiating mitochondrial biogenesis and metabolic reprogramming; post-exercise nutrient supply reverses this dominance, reactivating mTORC1 to rebuild structural and enzymatic capacity. When repeated across training cycles, this alternation generates oscillatory stability—a dynamic equilibrium between breakdown and synthesis that defines efficient adaptation [70,86].

**Figure 2:**
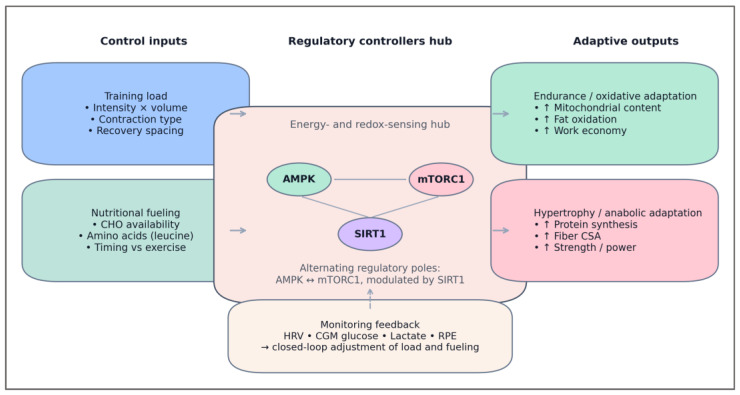
Core regulatory architecture of the Training–Fuel Coupling (TFC) model. Training load and nutritional fueling act as dual control inputs regulating molecular pathways (AMPK, mTORC1, and SIRT1) that determine adaptive outcomes in endurance and hypertrophy. Monitoring feedback (HRV, CGM, lactate) closes the regulatory loop, ensuring dynamic balance between energy stress and fuel availability. This diagram is conceptual and integrative in scope and is not intended to represent a fully parameterized quantitative model. Arrows indicate directional regulatory relationships and feedback interactions within the model.

The resulting control model is summarized schematically in Figure 2, which illustrates the dual-input, tri-controller structure of the Training–Fuel Coupling (TFC) system. It highlights how training load and nutritional fueling jointly modulate AMPK, mTORC1, and SIRT1 signaling to coordinate adaptive outcomes across energy stress and recovery phases.

As illustrated in Figure 2, the Training–Fuel Coupling framework operates as a self-regulating control system in which AMPK and mTORC1 act as alternating regulatory poles, with SIRT1 mediating their redox-dependent crosstalk. Through this dynamic interplay, training and nutrition jointly determine the polarity and magnitude of molecular signaling, translating energetic variability into functional remodeling. This structure forms the foundation for analyzing the system’s dynamic behavior.

**Figure 3:**
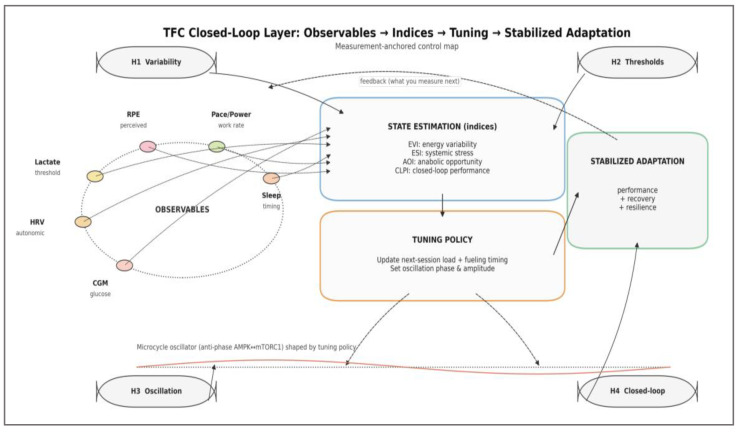
Measurement-anchored control map. Six routinely accessible observables—CGM (glucose), HRV (autonomic), Lactate (threshold), RPE (perceived), Pace/Power (work rate), and Sleep (timing)—inform STATE ESTIMATION (indices), which compresses signals into EVI (energy variability), ESI (systemic stress), AOI (adaptive oscillation), and CLPI (closed-loop performance). These indices parameterize the TUNING POLICY, which updates next-session load + fueling timing and sets oscillation phase & amplitude, shaping the Microcycle oscillator (anti-phase AMPK-mTORC1). STABILIZED ADAPTATION (performance + recovery + resilience) feeds back to subsequent OBSERVABLES, enabling iterative closed-loop regulation across the microcycle (H1 Variability, H2 Thresholds, H3 Oscillation, H4 Closed-loop). The map summarizes measurement-to-control logic and should be interpreted as a conceptual systems overlay rather than a validated operational algorithm. Colors distinguish functional modules, and arrows/dashed lines indicate directional influence and feedback relationships within the control loop.

**Figure 4:**
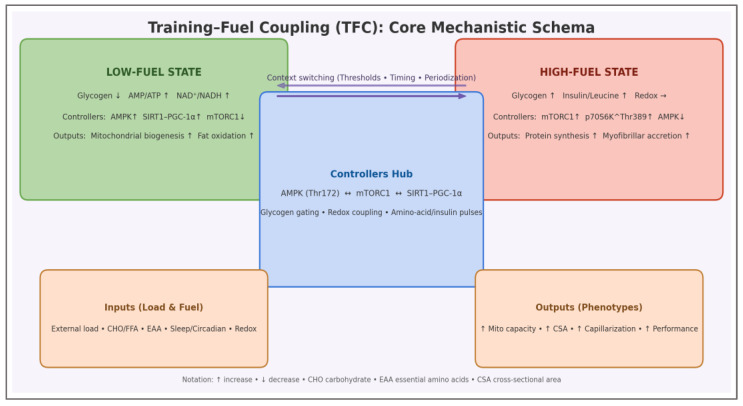
Core mechanistic schema of the Training–Fuel Coupling (TFC) framework. Low-fuel contexts (glycogen↓, AMP/ATP↑, NAD^+^/NADH↑) bias AMPK/SIRT1–PGC-1α and oxidative remodeling, whereas high-fuel contexts (glycogen↑, insulin/leucine↑) bias mTORC1/p70S6K and translational drive. Inputs (load and fuel) act through a controllers hub; context switching (thresholds, timing, periodization) flips signaling dominance and shapes adaptive outputs. The schema illustrates regulatory polarity and context-switching logic; it is not a complete representation of all signaling pathways.

Interpretative note on visual density— Figure 2 Figure 3 Figure 4 are intentionally information-dense because they consolidate multi-layered regulatory relationships (inputs, controllers, feedback, and adaptive outputs) into unified system schematics. They are designed as integrative conceptual maps rather than stepwise mechanistic diagrams. Accordingly, the figures should be read as structural overviews that summarize regulatory topology, not as exhaustive molecular pathway charts. Simplification was deliberately avoided to preserve coherence between energetic inputs, signaling polarity, and feedback structure within a single visual frame.

### 2.5. Analytical Deduction

The analytical deduction phase explored the internal logic of the Training–Fuel Coupling (TFC) model to derive the dynamic behaviors predicted by its structure. Once the regulatory architecture was defined, the system’s feedback interactions were analyzed qualitatively to determine how variations in training intensity and substrate availability propagate through molecular signaling pathways to produce adaptive outcomes. This reasoning followed a control-theoretic logic rather than computational simulation: the goal was to identify consistent behavioral patterns—emergent modes—resulting from the model’s structure and feedback polarity [87,88].

The analysis began by examining energy flux sensitivity, defined as the degree to which energetic inputs (training load and nutrient availability) shift the balance between AMPK and mTOR dominance. Under low-energy conditions, AMPK activation suppresses mTOR signaling, initiating a catabolic state that enhances oxidative capacity and stress tolerance. When the energetic context reverses, nutrient-driven activation of mTORC1 dominates, promoting anabolic repair and protein synthesis. The alternation of these states across repeated training cycles generates a self-regulating oscillation—an adaptive rhythm that improves system efficiency over time.

From this dynamic emerged the first major behavioral principle: energetic variability enhances adaptive efficiency (Hypothesis H1). Alternating between states of energy scarcity and abundance preserves the responsiveness of both signaling axes, preventing desensitization and enabling a wider range of physiological adjustments. Conversely, constant energetic context (e.g., chronically high carbohydrate or continuous low-glycogen training) leads to pathway saturation and adaptive rigidity.

The second behavior derived from the model concerns threshold regulation (H2). The interplay between AMPK activation thresholds and mTOR sensitivity defines a switching boundary that determines when the system transitions from catabolic to anabolic dominance. Small perturbations in training load or substrate level near this threshold produce disproportionately large shifts in signaling outcome, explaining the non-linear and often individualized responses observed in practice. This threshold behavior reflects the bistable nature of coupled feedback loops—once the system crosses a critical energetic boundary, regulatory dominance flips rapidly, generating distinct adaptive trajectories.

A third emergent property is oscillatory coupling (H3), describing the periodic alternation of signaling dominance across microcycles. When training and feeding stimuli are alternated systematically, AMPK and mTOR form an interlocked rhythm—each phase preparing the molecular landscape for the other. This oscillation produces cumulative adaptations greater than the sum of isolated responses, analogous to resonance in physical systems. The model predicts that the frequency and amplitude of these oscillations determine the rate and magnitude of adaptation: excessive frequency yields instability, while insufficient alternation results in plateau.

Finally, the system demonstrates closed-loop regulation (H4), a property whereby adaptive outcomes modify future system sensitivity. As mitochondrial expansion lowers energy stress, subsequent training bouts elicit weaker AMPK activation, shifting the system gradually toward anabolic readiness. Similarly, increased muscle mass raises metabolic demand, re-sensitizing AMPK in later cycles. This feedback closure explains the adaptive phase-shifting observed in periodized training, where metabolic efficiency evolves without external recalibration.

To operationalize H1–H4 in a form that is directly measurable, we summarize a measurement-anchored control map linking OBSERVABLES to STATE ESTIMATION (indices), TUNING POLICY, and STABILIZED ADAPTATION across the microcycle (Figure 3). In this overlay, observables are treated as pragmatic proxies for state estimation, and the feedback arrow encodes how stabilized adaptation updates what is measured next.

Figure 3 is intentionally complementary to the mechanistic architecture: it specifies the measurement-to-control pathway (observables → indices → tuning policy) and the closed-loop feedback (stabilized adaptation → observables) without redefining intracellular signaling detail. The subsequent subsections formalize these indices and the microcycle oscillator assumptions used for analytical deduction.

These deductions, summarized in figure above, transform the TFC model from a static framework into a predictive regulatory system, capable of explaining diverse training phenomena within a single logic structure. The four emergent behaviors—variability, threshold, oscillation, and closure—represent testable hypotheses linking molecular regulation to practical adaptation. Each describes a distinct dynamic regime through which the training–nutrition interaction shapes performance outcomes, providing the mechanistic rationale for the quantitative formalization and conceptual results presented in the subsequent sections.

### 2.6. Quantitative Formalization and Operationalization of the TFC Dynamics

To support conceptual clarity and hypothesis generation, the Training–Fuel Coupling framework introduces a set of composite indices intended to represent key regulatory dimensions of energetic control, including energetic variability, stress polarity, oscillatory timing, and feedback efficiency. These indices are presented as heuristic constructs designed to organize empirical observations and guide future experimental testing, rather than as empirically validated or operational metrics. Formal definitions, mathematical expressions, and illustrative thresholds are therefore provided in the Supplementary Materials.

### 2.7. Model Validation and Visualization

Conceptual and structural validation of the TFC framework was conducted at two complementary levels.

Internal validation confirmed that the regulatory architecture preserved established signaling polarities—AMPK as the energy-conserving feedback regulator, mTORC1 as the growth-promoting amplifier, and SIRT1 as the redox-sensitive modulator. This verification ensured the internal logical coherence of the dual-input, tri-controller design and the correct directionality of its feedback loops [89].

External validation examined the correspondence between model predictions and representative findings from exercise–nutrition research. Evidence from carbohydrate periodization, fasted versus fed training, and concurrent adaptation studies aligned with the predicted behaviors of the TFC framework, particularly the alternation of energetic dominance and the threshold-dependent transitions between catabolic and anabolic states [90].

Schematic visualization was employed throughout this process to consolidate the regulatory relationships and verify system coherence. This diagrammatic validation step ensured transparency between theoretical inference and physiological evidence, forming the methodological foundation for the analytical and empirical results [91].

## 3. Integrative Results of the Conceptual Framework

The conceptually corroborated Training–Fuel Coupling (TFC) framework delineates four emergent behaviors that describe how training and nutritional inputs interact to modulate adaptive outcomes. Unlike statistical results, these findings represent the mechanistic expressions of the system’s internal logic—patterns of regulation that arise from the interplay between energetic state and molecular control. Together, they explain how adaptation efficiency, threshold transitions, oscillatory dynamics, and feedback closure emerge from the same regulatory structure.

At the model level, the alternation between energetic scarcity and abundance generated a rhythmic modulation of AMPK and mTORC1 dominance, forming the basis of energetic variability. This property allowed the system to maintain sensitivity to both catabolic and anabolic stimuli, preventing pathway desensitization. Around this oscillating core, three additional regulatory behaviors appeared: a threshold mechanism that governs state transitions between energy stress and recovery; an oscillatory coupling pattern that produces phase-dependent enhancement optimization of adaptative efficiency; and a closed-loop feedback dynamic through which the outcomes of one cycle adjust the responsiveness of the next. Each of these emergent properties corresponds to one of the hypotheses formulated in the Introduction and derived analytically in the Methods.

The results are presented sequentially according to the system’s internal logic:

12. Energetic variability and adaptive efficiency (H1), describing how alternating metabolic contexts enhance long-term adaptation;
13. Threshold regulation of signaling dominance (H2), defining the energetic and molecular boundaries that separate catabolic from anabolic states;
14. Oscillatory coupling and periodized adaptation (H3), explaining the rhythm of signaling alternation across microcycles; and
15. Closed-loop regulation and feedback optimization (H4), illustrating how adaptive outcomes reshape future system sensitivity.

Each subsection integrates model-derived reasoning with empirical correspondence, linking mechanistic prediction to observed training phenomena. Collectively, these results position the TFC model as a generative conceptual framework that offers a structured interpretative lens for understanding adaptation dynamics and for formulating testable hypotheses across varied training and nutritional contexts.

The core mechanistic architecture of the Training–Fuel Coupling framework is summarized in Figure 4, mapping energetic context to signaling polarity (AMPK-mTORC1) and adaptive outputs.

This schematic consolidates the internal logic of the TFC model by mapping energetic context to regulatory polarity and phenotype formation. The two operational states—low-fuel and high-fuel—are defined by substrate availability and energy/redox charge, while the controllers hub formalizes the gating through AMPK–mTORC1–SIRT1/PGC-1α interactions. In this representation, training load and nutrient timing are not independent modifiers but levers that reposition the system across energetic thresholds, thereby determining whether remodeling is directed toward mitochondrial efficiency or myofibrillar accretion.

16. Practical Interpretation and Translational Scope

The practical relevance of the Training–Fuel Coupling (TFC) framework is discussed to illustrate how its internal logic may inform hypothesis-driven interpretations of training–nutrition interactions. These applied perspectives are not intended as prescriptive recommendations or evidence-based guidelines, but as conceptual extensions derived from the proposed model and from established mechanistic findings in the literature. Accordingly, their interpretation should be regarded as exploratory and context-dependent, serving primarily to generate testable translational hypotheses rather than to define standardized intervention protocols.

### 3.1. Energetic Variability and Adaptive Efficiency (H1)

Within the Training–Fuel Coupling (TFC) framework, adaptive efficiency emerges from the controlled alternation between energetic deficit and nutrient abundance. This behavior represents the system’s capacity to oscillate between AMPK-driven catabolism and mTOR-driven anabolism, a dynamic that enables simultaneous improvements in oxidative and structural performance traits. Rather than maximizing energy availability or minimizing stress, the model predicts that adaptation depends on variability—the capacity to move between metabolic extremes with appropriate timing and recovery. A conceptual overview of the Training–Fuel Coupling indices is provided in Table 1.

Mechanistically, AMPK activation during energy stress initiates mitochondrial biogenesis and enhances substrate oxidation efficiency. This response elevates endurance capacity but temporarily suppresses anabolic processes. Subsequent nutrient availability reverses the regulatory dominance: increased amino acid and insulin signaling activate mTORC1, promoting translational efficiency and tissue repair. When these contrasting states alternate rhythmically, the system preserves responsiveness across both metabolic axes. The oscillation functions as an adaptive amplifier, converting transient energetic fluctuations into cumulative performance gains.

In the model, this alternation produces an energy-to-adaptation resonance: successive low- and high-energy states stimulate distinct but complementary pathways, leading to progressive improvement without chronic overload. By analogy to control theory, energetic variability increases the system’s adaptive gain—its ability to respond proportionally to new stimuli rather than stabilizing prematurely. This explains why monotonous training–feeding patterns often yield plateaus: constant energy context drives desensitization of AMPK and mTOR signaling, reducing molecular plasticity.

Empirical data align closely with this prediction. Endurance studies show that alternating low- and high-glycogen sessions enhances markers of mitochondrial adaptation (PGC-1α, citrate synthase activity) compared with uniform carbohydrate intake. In resistance and mixed modalities, alternating fasted and fed sessions increases both oxidative efficiency and lean mass, demonstrating that metabolic diversity reinforces adaptive potential. The model interprets these findings not as conflicting results but as evidence of the same principle—adaptation is optimized when energy availability fluctuates within physiologically manageable bounds.

Practically, energetic variability provides the mechanistic rationale for strategies such as “train-low, recover-high,” “sleep-low,” or mixed substrate periodization. These approaches exploit the oscillatory logic of the TFC model, deliberately modulating energetic context to maintain high signaling sensitivity. The model predicts an optimal frequency of alternation—frequent enough to prevent pathway saturation, but separated by sufficient recovery to avoid cumulative stress. Excessive variability disrupts regulatory synchronization, while insufficient variability induces stagnation.

In summary, Hypothesis 1 identifies energetic variability as a core determinant of adaptive efficiency. Through controlled alternation between AMPK and mTOR dominance, the TFC model transforms metabolic stress into a constructive signal, integrating endurance and hypertrophic processes within a single regulatory cycle. This principle establishes the foundation for subsequent system behaviors, in which threshold dynamics, oscillatory coupling, and feedback regulation refine and extend this adaptive logic.

The experimental validation logic associated with Hypothesis H1 is summarized in Supplementary Table S2.

This hypothesis establishes the contextual foundation of the TFC model, linking substrate-dependent signaling to differential adaptive outcomes under identical external workloads. By framing glycogen status, redox balance, and nutrient co-availability as dynamic regulators of molecular bias, it defines the first empirical layer of energetic control. The corresponding Validation Landscape integrates these dimensions into a unified experimental view, detailing how metabolic context, signaling reciprocity, and redox variability define the conceptual structure of energetic control.

### 3.2. Threshold Regulation of Signaling Dominance (H2)

The Training–Fuel Coupling (TFC) model predicts that adaptation transitions are governed not by gradual changes but by threshold-dependent switches in signaling dominance. Within the regulatory architecture, AMPK and mTORC1 operate as opposing control nodes separated by an energetic boundary that determines which pathway prevails at a given moment. This threshold reflects the point at which cellular energy charge, expressed through AMP/ATP and NAD^+^/NADH ratios, reaches a level sufficient to flip the system from a catabolic to an anabolic state. The model therefore treats adaptation as a sequence of controlled state transitions rather than a continuous scaling process.

Mechanistically, the threshold emerges from reciprocal inhibition between AMPK and mTORC1. When ATP depletion and increased AMP concentration activate AMPK, phosphorylation of TSC2 and Raptor suppresses mTORC1 signaling, stabilizing the system in a low-energy, oxidative mode. As substrate availability restores energy charge, this inhibition is relieved: rising insulin and amino acid levels activate Akt and Rag GTPases, re-engaging mTORC1 and shifting the system toward anabolic reconstruction. The moment of transition—the energetic threshold—defines a nonlinear inflection where small perturbations in fuel availability produce large shifts in regulatory dominance.

This behavior explains why individual responses to training and nutrition often diverge despite similar external loads. Athletes operating near this boundary may exhibit high variability in adaptation, as slight differences in glycogen stores or feeding timing determine which side of the threshold the system occupies. The model thus reinterprets the so-called “interference effect” of concurrent training: it is not a conflict between endurance and strength stimuli, but a misalignment in energetic state relative to the signaling threshold. When training and nutrition are synchronized to cross this threshold strategically, catabolic and anabolic signals become sequential rather than competitive.

Empirical findings support this interpretation. Studies show that post-exercise protein ingestion during low-glycogen recovery attenuates mTORC1 activation, while the same intake under glycogen-restored conditions amplifies it. Similarly, resistance sessions performed under mild energy deficit enhance oxidative enzyme activity but blunt hypertrophic signaling, reflecting a shift below the energetic threshold. These outcomes align with the TFC model’s prediction that adaptation follows a bistable logic: once a critical metabolic boundary is crossed, the system switches decisively between regulatory modes.

From a practical standpoint, threshold regulation implies that small adjustments in carbohydrate or protein timing can yield disproportionate effects on adaptation quality. It highlights the need for precision in periodized nutrition—manipulating energy state not only by magnitude but by timing relative to the signaling threshold. This view reframes metabolic control as a problem of regulatory positioning, in which the athlete’s metabolic state must oscillate across the boundary to sustain adaptive responsiveness.

In summary, Hypothesis 2 identifies the existence of an energetic threshold that governs the transition between catabolic and anabolic dominance. This threshold-driven behavior introduces nonlinearity into the adaptation process and provides a mechanistic explanation for interindividual variability in training outcomes. Within the TFC framework, it represents the system’s internal switch—an energy-dependent logic gate that coordinates the alternation of regulatory control across successive training–nutrition cycles.

The experimental logic and validation framework associated with Hypothesis H2 are summarized in Supplementary Table S3.

This hypothesis formalizes the concept of an energetic boundary that determines the polarity of molecular signaling. It describes how small shifts in energy charge and redox potential can flip the regulatory dominance between AMPK and mTORC1, acting as a bistable metabolic switch. The associated Validation Landscape articulates this mechanism across structural levels—from substrate thresholds and energetic stress indices to experimentally observable phosphorylation ratios—mapping the path from theoretical gating to empirically interpretable adaptive patterns.

### 3.3. Oscillatory Coupling and Periodized Adaptation (H3)

The Training–Fuel Coupling (TFC) framework interprets adaptation as a rhythmic process in which catabolic and anabolic states alternate in predictable cycles. This oscillatory coupling between AMPK and mTORC1 signaling represents the physiological foundation of periodized training, translating molecular timing into functional performance gains. Rather than treating recovery as a passive return to baseline, the model positions it as the complementary half of the adaptive rhythm—a phase that completes, not interrupts, the training signal.

**Figure 5:**
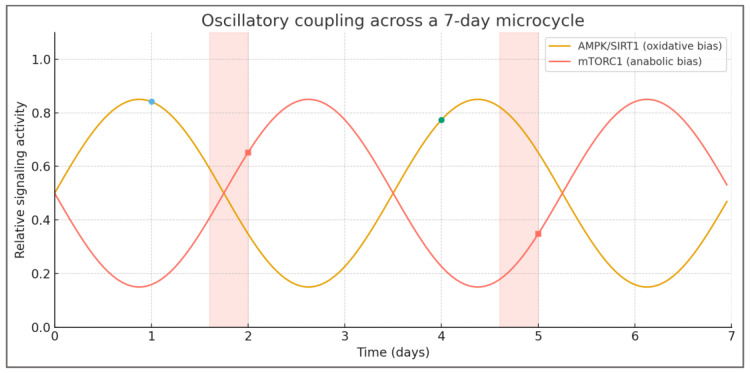
Oscillatory coupling across a 7-day microcycle. Colored dots mark representative session types within the microcycle, and shaded areas indicate high-fuel windows preceding resistance sessions.

In the integrated control system, AMPK and mTORC1 form a negative feedback pair. Activation of one pathway inherently suppresses the other, creating a self-sustaining oscillation around an energetic equilibrium point. During the stress phase, energy depletion triggers AMPK, enhancing mitochondrial efficiency and substrate oxidation. As recovery and feeding restore energy availability, mTORC1 activation dominates, stimulating protein synthesis and tissue repair. Once energetic abundance stabilizes, anabolic signaling declines, gradually re-sensitizing AMPK and setting the stage for the next stress phase. Each oscillation thus refines system responsiveness—AMPK improves efficiency under load, while mTORC1 strengthens capacity during recovery. This alternating dominance creates a rhythmic sequence that can be visualized across a typical training week, as shown in Figure 5.

Low-fuel endurance sessions align with AMPK/SIRT1 peaks, whereas brief high-fuel windows precede fed resistance sessions, coinciding with mTORC1 dominance. This arrangement maintains metabolic sensitivity by alternating oxidative and anabolic contexts across the microcycle.

This cyclical alternation produces emergent stability through rhythmic imbalance. The system does not seek a static homeostasis but rather an adaptive rhythm, oscillating between states of stress and replenishment. Such behavior mirrors other biological control systems—from circadian regulation to endocrine feedback—where efficiency and resilience arise from controlled fluctuation, not constancy. In the TFC model, the amplitude of oscillation represents the degree of metabolic challenge, while its frequency reflects training density and nutritional timing. Optimal adaptation occurs when oscillations are neither too shallow nor too steep, maintaining synchronization between metabolic stress and recovery.

Empirical observations across endurance, resistance, and mixed training support this logic. Studies of carbohydrate periodization show that alternating glycogen-depleted and glycogen-restored sessions enhances both oxidative enzymes and performance capacity. In resistance training, alternating fasted and fed sessions yields superior adaptation compared to uniform feeding protocols. These findings correspond to the model’s prediction that oscillatory coupling—rather than constant energy state—maximizes signaling sensitivity and adaptive output. The alternation of AMPK and mTORC1 dominance thus functions as a biological metronome, coordinating energy flux with molecular remodeling.

At the practical level, oscillatory coupling provides the mechanistic rationale for structured periodization. Training cycles that deliberately modulate substrate availability (for example, high-intensity sessions under low glycogen followed by nutrient-rich recovery) replicate the natural oscillatory pattern of metabolic signaling. The TFC framework interprets successful periodization not as an art of scheduling but as the engineering of metabolic resonance—the tuning of energetic rhythm to the adaptive capacity of the organism. Disruption of this rhythm, through monotonous training or chronic nutritional excess, dampens oscillatory amplitude and leads to stagnation or maladaptation.

In summary, Hypothesis 3 defines adaptation as a product of rhythmic coupling between opposing regulatory pathways. The oscillatory behavior of the AMPK–mTORC1 system transforms metabolic stress into a structured pattern of activation and recovery, aligning molecular timing with functional periodization. Within the TFC model, this rhythmic alternation is the core dynamic that maintains adaptive momentum across cycles, ensuring that each phase of stress and recovery potentiates the next.

The experimental validation landscape for oscillatory coupling and periodized adaptation (Hypothesis H3) is detailed in Supplementary Table S4.

This hypothesis extends the TFC framework into the temporal domain, proposing that deliberate oscillation between catabolic and anabolic phases stabilizes adaptive output over time. It unites molecular rhythm, nutritional synchronization, and mesocycle design into a single systems perspective of training adaptation. The Validation Landscape translates these ideas into testable constructs, outlining parameters of oscillatory coherence, adaptive amplitude, and synchronization fidelity that characterize how metabolic rhythm shapes performance plasticity.

### 3.4. Closed-Loop Regulation and Feedback Optimization (H4)

The Training–Fuel Coupling (TFC) model ultimately converges toward a closed-loop form of regulation, in which each adaptive outcome modifies the system’s future responsiveness. This behavior represents the self-organizing capacity of the human metabolic network—a process through which adaptation refines its own conditions. Unlike open systems, where each stimulus is treated as independent, the TFC framework assumes recursive adaptation: every training–nutrition cycle alters the internal parameters governing subsequent regulation.

Mechanistically, closed-loop regulation emerges from reciprocal feedback between structural and energetic adaptation. Mitochondrial expansion resulting from AMPK activation reduces future energy stress for a given workload, lowering the threshold for mTORC1 engagement during recovery. Conversely, hypertrophic remodeling driven by mTORC1 increases metabolic demand, re-sensitizing AMPK activation in later sessions. This bidirectional adjustment forms a metabolic feedback circuit that progressively narrows the gap between energy supply and energy cost, thereby improving system stability. Over repeated cycles, the feedback loop tunes itself—reducing oscillatory error while preserving rhythmic adaptability.

This property of feedback optimization explains why adaptation follows diminishing returns: as efficiency improves, the relative perturbation produced by a given training stimulus decreases. In the model, the system approaches a quasi-stable attractor—an adaptive equilibrium defined not by stasis, but by minimal error between energy input, recovery capacity, and performance output. Such behavior parallels control processes in engineered systems, where adaptive algorithms iteratively refine performance through feedback minimization. Biologically, this closed-loop tuning manifests as improved metabolic economy, enhanced recovery kinetics, and reduced fatigue for equivalent workloads.

Empirical evidence supports the existence of this self-tuning mechanism. Longitudinal studies reveal that trained individuals exhibit blunted AMPK activation and faster mTORC1 rebound compared to novices, reflecting a recalibrated energetic sensitivity. Similarly, repeated exposure to periodized training cycles reduces the amplitude but increases the efficiency of metabolic oscillations—a hallmark of optimized feedback regulation. These findings reinforce the TFC model’s interpretation of adaptation as a feedback process rather than a linear accumulation of effects.

From an applied perspective, recognizing adaptation as a closed-loop system redefines training progression. It shifts focus from imposing greater stress to refining system responsiveness—using recovery, nutrient timing, and load variation to maintain optimal feedback gain. Effective periodization therefore becomes a matter of managing sensitivity, not intensity. In this view, training ceases to be an external input and becomes an ongoing dialog between system capacity and energetic context.

In summary, Hypothesis 4 establishes that adaptation within the TFC framework is governed by closed-loop feedback optimization. Each phase of training and recovery modifies the parameters controlling subsequent adaptation, creating a self-tuning cycle that stabilizes efficiency while preserving responsiveness. This behavior completes the regulatory circuit of the model, demonstrating how metabolic systems transform fluctuating energy states into sustained performance through recursive adjustment and feedback integration.

The systems-level validation framework for closed-loop regulation and feedback optimization (Hypothesis H4) is provided in Supplementary Table S5.

This hypothesis operationalizes the TFC model within a feedback paradigm, where internal physiological signals actively shape substrate intake and training behavior. It defines adaptation as an emergent property of recurrent feedback—where sensing, adjustment, and efficiency evolve together toward homeostatic optimization. The following Validation Landscape presents the systems-level blueprint for testing this principle, integrating physiological telemetry, and feedback logic into a coherent framework of adaptive self-regulation.

### 3.5. Validation Model Summary

The four TFC hypotheses collectively define a mechanistic framework that can be validated through tiered experimentation.

At the molecular level, validation involves quantifying reciprocal phosphorylation patterns (↑*p*-AMPK^Thr172, ↑*p*-ACC^Ser79, ↓*p*-p70S6K^Thr389) across energetic contexts defined by physiologically relevant ranges of muscle glycogen, AMP/ATP ratio, and NAD^+^/NADH redox balance.

At the functional level, validation requires integrated assessment of mitochondrial and contractile adaptations—indices of mitochondrial, contractile, and performance adaptation, and time-trial performance—to confirm oscillatory synergy predicted by H3.

At the systems level, feedback-based monitoring (RPE×[La], ΔHRV, Δglucose) provides real-time markers for the closed-loop adaptation process described in H4.

Together, these validation pathways form a coherent experimental model:

17. Contextual gating (H1–H2) → mechanistic confirmation via phosphorylation assays and metabolic profiling.
18. Oscillatory adaptation (H3) → mesocycle interventions demonstrating dual-pathway enhancement.
19. Feedback optimization (H4) → longitudinal monitoring of adaptive efficiency and stability.

This tiered validation model bridges theoretical deduction and empirical testing, allowing the Training–Fuel Coupling architecture to evolve from a conceptual construct into a falsifiable, experimentally grounded physiological framework.

Collectively, these validation tiers define a coherent conceptual architecture for the Training–Fuel Coupling framework, integrating molecular signaling, functional adaptation, and systems-level regulation within a unified physiological logic.

An integrated experimental validation roadmap linking Hypotheses H1–H4 across molecular, functional, and systems levels is presented in Supplementary Table S6.

Applied decision matrices aligning training objectives, energetic contexts, and adaptive outcomes derived from the Training–Fuel Coupling framework are provided in Supplementary Table S7.

### 3.6. Testable Predictions

The Training–Fuel Coupling (TFC) model generates a series of experimentally testable hypotheses:

20. Reciprocal signaling amplitude—within alternating low-fuel and high-fuel microcycles, AMPK and mTORC1 phosphorylation amplitudes will oscillate in anti-phase (*p*-AMPK^Thr172↑ when glycogen is low; *p*-p70S6K^Thr389↑ after refueling).
21. Threshold-dependent switching—metabolic dominance will shift when glycogen content passes a critical energetic range, reflected in inverted correlations between AMPK and mTORC1 activity.
22. SIRT1 modulation of cross-talk—NAD^+^/NADH oscillations will parallel AMPK phase activation and predict mTORC1 re-sensitization after refueling.
23. Performance-phase coupling—functional adaptations will follow the same periodicity—improved oxidative efficiency during AMPK phases, enhanced muscle hypertrophy and strength during mTORC1 phases.
24. Systems coherence—derived composite indices (Energetic Variability Index, Adaptive Oscillation Index, Closed-Loop Performance Index) will covary with molecular and performance oscillations across repeated microcycles.

Validation of these predictions requires longitudinal, multi-omics designs combining muscle biopsy, continuous glucose monitoring, and performance testing across controlled train-low/train-high rotations.

Exemplary microcycle configurations illustrating how the Training–Fuel Coupling framework can be translated into practice are provided in Supplementary Table S8.

Together, these testable predictions illustrate how the Training–Fuel Coupling framework links molecular regulation to adaptive dynamics through controlled energetic variability, temporal coordination, and feedback integration.

## 4. Discussion

The Training–Fuel Coupling (TFC) model offers a mechanistic synthesis that unites molecular, nutritional, and training evidence within a single regulatory framework. Its principal contribution lies in redefining adaptation as a system behavior—an emergent property of dynamic control loops linking energy availability, signaling balance, and functional remodeling. Through this lens, the long-debated divide between “train-low” metabolic stress and “feed-high” recovery can be interpreted not as a contradiction but as a sequence of regulatory states that, when properly alternated, optimize the rhythm of adaptation [92,93,94,95].

Integration with Previous Research—Previous studies have independently described the benefits of metabolic stress and nutrient abundance yet often treated them as competing stimuli. Endurance literature highlights the mitochondrial benefits of low-glycogen training, while resistance research emphasizes protein synthesis under nutrient-rich conditions. The TFC framework reconciles these findings by proposing that both processes are phases of the same adaptive oscillation. Similar oscillatory behavior has been documented in cellular systems such as AMPK–mTOR feedback, circadian energy sensing, and hormonal regulation, suggesting that rhythm-based adaptation is a conserved biological strategy [96,97,98].

The concept also aligns with emerging perspectives in molecular exercise physiology that view training adaptation as a nonlinear process governed by thresholds, sensitivity, and feedback. Studies using transcriptomic and proteomic profiling confirm that repeated energy fluctuations elicit cyclical activation of metabolic and anabolic gene networks, mirroring the alternating dominance predicted by the model. Moreover, the self-tuning feedback mechanism proposed in the TFC framework parallels observations from long-term training studies, where improved fitness is accompanied by reduced signaling amplitude but enhanced response precision—a hallmark of closed-loop regulation [99,100,101,102].

Interpretation of Findings Relative to Hypotheses—The four hypotheses derived from the analytical phase describe complementary aspects of this self-organizing system. Hypothesis 1—energetic variability enhances adaptive efficiency—is supported by consistent evidence showing superior outcomes from alternating metabolic conditions. Hypothesis 2—threshold regulation—finds analogs in bistable molecular systems, where minor changes in substrate level induce large signaling shifts, explaining individual variability in training responses. Hypothesis 3—oscillatory coupling—connects directly to the principles of periodized training, establishing a molecular basis for timing-dependent adaptation. Finally, Hypothesis 4—closed-loop optimization—extends these ideas into a long-term perspective, showing how adaptation progressively recalibrates its own sensitivity to energetic stimuli. Together, these behaviors form a continuum from acute metabolic signaling to chronic system stabilization.

Broader Implications—By articulating adaptation as a self-regulating control process, the TFC model bridges reductionist and applied perspectives in sport science. It provides a theoretical foundation for nutritional periodization strategies and hybrid training designs, offering hypothesis-driven guidance on when to emphasize metabolic stress versus recovery nutrition. Beyond athletic performance, the same logic may apply to clinical contexts where energy regulation and tissue remodeling are critical, including metabolic syndrome, sarcopenia, or rehabilitation following immobilization. In such settings, managing the timing of energetic inputs could restore adaptive plasticity without excessive physiological strain [103,104,105,106].

Boundaries and Safety Considerations—The practical implementation of the Training–Fuel Coupling (TFC) framework requires oscillation within physiological limits of energy availability and recovery. In this framework, energy availability refers to the instantaneous metabolic state, whereas energetic variability describes the regulated alternation of energy availability across training cycles. The distinction between functional energetic variability and chronic energy deficiency is essential to prevent maladaptation.

Controlled “train-low” strategies are effective only when glycogen availability fluctuates within a safe range and protein intake remains sufficient to support post-exercise mTORC1 reactivation. Sustained depletion beyond these limits is associated with documented suppression of anabolic signaling, compromised endocrine and immune balance, and an increased risk of relative energy deficiency in sport (RED-S) [107,108].

Oscillatory amplitude should therefore be modulated by context, as sex, training phase, and nutritional history influence the safe energetic bandwidth. Female and adolescent athletes, as well as periods of elevated training load, require narrower fluctuations and closer monitoring. Continuous feedback through HRV, CGM, and subjective wellness indices may help identify early deviations from adaptive zones [109,110,111].

In this view, the TFC concept emphasizes metabolic variability as a regulated adaptive process, not as voluntary deprivation. Taken together, these considerations clarify that Training–Fuel Coupling is not intended as a prescriptive nutritional protocol but as a conceptual framework for understanding how energy availability and nutrient timing shape adaptive signaling. The model does not advocate chronic energy restriction or fixed feeding strategies; rather, it provides a systems-level rationale for aligning metabolic stress and recovery nutrition within safe physiological boundaries. From this perspective, maladaptive outcomes such as Relative Energy Deficiency in Sport (RED-S) may arise when regulatory oscillation and recovery integration fail, rather than being inevitable consequences of metabolic stress per se.

Conflicting evidence and biological trade-offs—Although mechanistic studies frequently report amplified oxidative signaling under low-glycogen conditions, the performance-level evidence for long-term carbohydrate periodization remains heterogeneous. Several systematic reviews and controlled trials report mixed or null effects on functional outcomes (e.g., time-trial performance or steady-state capacity) despite molecular signatures consistent with enhanced remodeling [29,62,66]. This divergence highlights an important distinction: acute signaling and transcriptomic responses do not necessarily translate into superior long-term performance, particularly when training quality, recovery, and total energy availability are compromised. In parallel, repeated exposure to low-energy availability may impose biological costs—endocrine disruption, immune perturbation, impaired sleep/recovery, and increased risk of Relative Energy Deficiency in Sport (RED-S)—especially when “train-low” sessions are layered onto chronically constrained intake [106,107]. Within the TFC perspective, these trade-offs are interpreted as constraints on oscillation amplitude and frequency: adaptive benefits are hypothesized to occur within a bounded “safe bandwidth,” whereas chronic or excessive deficits may shift the system toward maladaptation. This boundary framing motivates future empirical tests that jointly quantify performance, molecular signaling, and health-related markers across different oscillation patterns, rather than treating carbohydrate restriction as uniformly beneficial.

Limitations and Scope—The Training–Fuel Coupling (TFC) framework is a conceptual synthesis rather than an empirical dataset. Its logic is derived from established signaling interactions, physiological observations, and theoretical integration. Therefore, the numerical thresholds indicated for glycogen or redox status represent context-dependent estimates, not universal constants. The oscillatory patterns proposed are model-derived abstractions that require experimental quantification under varied training and nutritional settings. Moreover, the framework focuses on skeletal muscle energetics and nutrient-sensitive signaling as a defined analytical scope, while other regulatory levels—endocrine, immune, neural, and behavioral—are acknowledged but not explicitly modeled. Within this defined analytical scope, the AMPK–mTOR axis is treated as a dominant integrative regulator of energetic adaptation, but not as an exclusive determinant of exercise-induced physiological remodeling. Accordingly, the explanatory reach of the TFC framework should be understood as structurally integrative rather than universally predictive, operating within clearly defined biological and conceptual boundaries. Future studies should test the model’s predictions across different populations, sport modalities, and time scales to refine its generalizability.

Biological scope beyond AMPK-mTOR—While AMPK–mTORC1 provides a parsimonious integrative axis for nutrient-sensitive energetic and anabolic regulation, exercise adaptation is also shaped by parallel signaling routes (e.g., p38 MAPK/ERK and CaMKII/calcineurin-related pathways) and endocrine regulation (including HPA-axis dynamics and catecholamine/steroid signaling) that influence substrate flux, inflammation, and gene expression. In addition, responses are not uniform across tissues that govern systemic energy availability (liver glycogen turnover, adipose lipolysis, gut absorption, and autonomic control), nor across muscle fiber types (type I vs. type II), which differ in metabolic profile and remodeling sensitivity. The present TFC framework intentionally abstracts these layers to focus on skeletal muscle energetics and nutrient-sensitive gating; therefore, it should be interpreted as describing a dominant regulatory motif rather than a complete multiscale model. Future extensions could incorporate tissue/fiber-type parameterization and hormonal constraints as higher-level modulators of the core energetic oscillation.

Within these limitations, the Training–Fuel Coupling (TFC) model operates under explicit assumptions and yields testable predictions. It assumes that variation in energy availability and nutrient timing meaningfully modulates the balance between AMPK- and mTOR-dominated signaling, and that adaptive outcomes arise from repeated oscillations between energetic stress and recovery. The model is not intended to apply to contexts with chronically stable energy availability or where adaptation is dominated by non-metabolic constraints. Importantly, TFC proposes that systematic manipulation of energy availability and nutrient timing under matched training loads may lead to divergent adaptive signaling and functional outcomes; these predictions can be empirically tested using controlled training interventions in which training load is held constant while energy availability and nutrient timing are systematically varied, with molecular and functional outcomes compared across conditions.

Future Directions—The TFC framework should be formalized into quantitative models that simulate adaptive dynamics under varying training and nutritional schedules. Integrating wearable sensor data, metabolomics, and machine learning could allow the real-time estimation of energetic state and regulatory phase, enabling individualized periodization. Experimental validation of oscillatory coupling—via molecular markers of AMPK/mTOR activity across repeated cycles—would test the predictive accuracy of the framework. Further, longitudinal studies exploring how closed-loop adaptation shapes long-term efficiency may reveal how metabolic systems approach steady-state performance without plateauing.

Conceptual Significance—In conceptual terms, the TFC model reframes human adaptation as an intelligent process of energetic governance, a dynamic interplay between stress and recovery, scarcity and abundance, catabolism and anabolism. It captures the essence of biological resilience: not merely resistance to change, but the capacity to transform fluctuation into structure. By coupling mechanistic precision with ecological relevance, the framework offers a unified language for interpreting training, metabolism, and nutrition as elements of a single adaptive continuum. In doing so, it positions metabolic oscillation as both a mechanism and a metaphor for sustainable performance and systemic balance.

## 5. Conclusions

This narrative review with conceptual synthesis introduces Training–Fuel Coupling (TFC) as a systems-level, hypothesis-generating framework for examining how exercise and nutrition interact in the regulation of adaptation. By framing training and feeding as coordinated control inputs within a dynamic regulatory network, the model integrates previously isolated findings across molecular, metabolic, and functional domains. The resulting framework conceptualizes adaptation not as the accumulation of stimuli but as the rhythmic alternation of energetic states that may drive efficiency through controlled instability.

Across its four emergent behaviors—variability, threshold, oscillation, and feedback—the TFC model articulates a coherent regulatory logic underlying human adaptability: responsiveness sustained through contrast, precision achieved through fluctuation, and stability maintained through feedback. From this perspective, apparent contradictions between endurance- and strength-oriented paradigms can be interpreted as differences in energetic timing and recovery alignment rather than as incompatible training principles.

By integrating molecular sports nutrition with energy availability and chrono-nutrition principles, the Training–Fuel Coupling framework proposes performance optimization as a self-tuning process linking cellular signaling to whole-body adaptation. Accordingly, TFC should be interpreted as a conceptual synthesis intended to organize existing evidence, clarify regulatory logic, and guide future empirical investigation, rather than as a definitive or empirically validated theory of training–nutrition interaction.

## Figures and Tables

**Table 1:** Conceptual overview of Training–Fuel Coupling (TFC) indices and their regulatory interpretation.

Index	Regulatory Dimension Captured	Conceptual Meaning Within TFC	Dominant Physiological Signals	Adaptive Role (Interpretative)
**Energetic** **Variability** **Index (EVI)**	Energetic state variability	Describes the degree of alternation between energy-deficient and energy-replete states across successive training sessions	Fluctuations in glucose availability, autonomic variability (HRV), session-to-session training context	Preserves signaling sensitivity and metabolic plasticity by preventing chronic dominance of a single energetic state
**Energetic** **Stress** **Index (ESI)**	Stress polarity	Represents the relative balance between catabolic energetic stress and anabolic recovery states	Lactate responses, autonomic suppression, post-exercise glucose dynamics	Indicates whether the system is biased toward adaptation-driving stress or toward recovery-driven restoration
**Adaptive** **Oscillation** **Index (AOI)**	Temporal coordination	Captures the rhythmic alternation between catabolic (AMPK-dominant) and anabolic (mTOR-dominant) signaling phases across training microcycles	Sequencing of stress- and recovery-dominant sessions, temporal alignment of fueling and load	Reflects the coherence of metabolic oscillation underlying efficient long-term adaptation
**Closed-Loop** **Performance** **Index (CLPI)**	Feedback efficiency	Describes how effectively physiological feedback links energetic input, recovery, and performance stability	Variability of session quality, recovery markers, consistency of performance output	Represents the efficiency of self-regulation and adaptive stabilization over repeated training cycles

Notes. The indices summarized here describe core regulatory dimensions of the Training–Fuel Coupling framework, capturing variability, stress polarity, oscillatory coordination, and feedback efficiency across training–nutrition cycles. They are intended to support conceptual interpretation of adaptive regulation rather than to represent fixed physiological measures or prescriptive thresholds.

## Data Availability

No new datasets were generated or analyzed in this narrative review. All information is derived from published sources cited in the References. Data are contained within the article and its Supplementary Materials.

## Supplementary Materials

The following supporting information can be downloaded at: https://www.mdpi.com/article/10.3390/nu18040693/s1, Table S1: Practical Biomarkers of Energetic State and TFC Proxy Indices; Table S2: Validation Landscape for H1—Energetic Variability and Adaptive Efficiency. Experimental design variables, signaling markers, and validation priorities defining substrate-dependent adaptive modulation; Table S3: Validation Landscape for H2—Threshold Regulation of Signaling Dominance. Experimental framework for quantifying energetic boundaries, signaling polarity, and bistable metabolic transitions; Table S4: Validation Landscape for H3—Oscillatory Coupling and Periodized Adaptation. Integrated experimental parameters linking metabolic rhythm, training periodization, and composite adaptive gain; Table S5: Validation Landscape for H4—Closed-Loop Regulation and Feedback Optimization. Systems-level framework for testing dynamic feedback, performance stability, and adaptive efficiency; Table S6: Experimental Validation Model for the Training–Fuel Coupling (TFC) Framework; Table S7: Training–Fuel Coupling (TFC) Decision Matrix—Linking Training Goals, Energetic Context, and Adaptive Outcomes; Table S8: Exemplary Microcycles Translating the Training–Fuel Coupling (TFC) Framework into Practice. References [112,113,114,115,116,117,118] are cited in the supplementary materials.

## References

[1] Coffey V.G., Hawley J.A. (2007). The molecular bases of training adaptation. Sports Med..

[2] Hawley J.A., Burke L.M., Phillips S.M., Spriet L.L. (2011). Nutritional modulation of training-induced skeletal muscle adaptations. J. Appl. Physiol..

[3] Egan B., Zierath J.R. (2013). Exercise metabolism and the molecular regulation of skeletal muscle adaptation. Cell Metab..

[4] Hawley J.A., Hargreaves M., Joyner M.J., Zierath J.R. (2014). Integrative biology of exercise. Cell.

[5] Atherton P.J., Smith K. (2012). Muscle protein synthesis in response to nutrition and exercise. J. Physiol..

[6] Hardie D.G., Ross F.A., Hawley S.A. (2012). AMPK: A nutrient and energy sensor that maintains energy homeostasis. Nat. Rev. Mol. Cell Biol..

[7] Gwinn D.M., Shackelford D.B., Egan D.F., Mihaylova M.M., Mery A., Vasquez D.S., Turk B.E., Shaw R.J. (2008). AMPK phosphorylation of raptor mediates a metabolic checkpoint. Mol. Cell.

[8] Fujii T., Kobayashi K., Kaneko M., Osana S., Tsai C.-T., Ito S., Hata K. (2024). RGM Family Involved in the Regulation of Hepcidin Expression in Anemia of Chronic Disease. Immuno.

[9] Inoki K., Zhu T., Guan K.-L. (2003). TSC2 mediates cellular energy response to control cell growth and survival. Cell.

[10] Jäger S., Handschin C., St-Pierre J., Spiegelman B.M. (2007). AMP-activated protein kinase (AMPK) action in skeletal muscle via direct phosphorylation of PGC-1α. Proc. Natl. Acad. Sci. USA.

[11] Hargreaves M., Spriet L.L. (2018). Exercise Metabolism: Fuels for the Fire. Cold Spring Harb. Perspect. Med..

[12] Kelley D.E., Mandarino L.J. (2000). Fuel selection in human skeletal muscle in insulin resistance: A reexamination. Diabetes.

[13] Mănescu A.M., Hangu S.Ș., Mănescu D.C. (2025). Nutritional Supplements for Muscle Hypertrophy: Mechanisms and Morphology—Focused Evidence. Nutrients.

[14] Galgani J.E., Moro C., Ravussin E. (2008). Metabolic flexibility and insulin resistance. Am. J. Physiol. Endocrinol. Metab..

[15] Goodpaster B.H., Sparks L.M. (2017). Metabolic Flexibility in Health and Disease. Cell Metab..

[16] McBride A., Ghilagaber S., Nikolaev A., Hardie D.G. (2009). The glycogen-binding domain on the AMPK beta subunit allows the kinase to act as a glycogen sensor. Cell Metab..

[17] Hansen A.K., Fischer C.P., Plomgaard P., Andersen J.L., Saltin B., Pedersen B.K. (2005). Skeletal muscle adaptation: Training twice every second day vs. training once daily. J. Appl. Physiol..

[18] Yeo W.K., Paton C.D., Garnham A.P., Burke L.M., Carey A.L., Hawley J.A. (2008). Skeletal muscle adaptation and performance responses to once a day versus twice every second day endurance training regimens. J. Appl. Physiol..

[19] Psilander N., Frank P., Flockhart M., Sahlin K. (2013). Exercise with low glycogen increases PGC-1α gene expression in human skeletal muscle. Eur. J. Appl. Physiol..

[20] Knuiman P., Hopman M.T.E., Mensink M. (2015). Glycogen availability and skeletal muscle adaptations with endurance and resistance exercise. Nutr. Metab..

[21] Smiles W.J., Ovens A.J., Kemp B.E., Galic S., Petersen J., Oakhill J.S. (2024). New developments in AMPK and mTORC1 cross-talk. Essays Biochem..

[22] White J.P. (2021). Amino Acid Trafficking and Skeletal Muscle Protein Synthesis: A Case of Supply and Demand. Front. Cell Dev. Biol..

[23] D’Hulst G., Masschelein E., De Bock K. (2022). Resistance exercise enhances long-term mTORC1 sensitivity to leucine. Mol. Metab..

[24] Ely I.A., Phillips B.E., Smith K., Wilkinson D.J., Piasecki M., Breen L., Larsen M.S., Atherton P.J. (2023). A focus on leucine in the nutritional regulation of human skeletal muscle metabolism in ageing, exercise and unloading states. Clin. Nutr..

[25] Ferrando A.A., Wolfe R.R., Hirsch K.R., Church D.D., Kviatkovsky S.A., Roberts M.D., Stout J.R., Gonzalez D.E., Sowinski R.J., Kreider R.B. (2023). International Society of Sports Nutrition Position Stand: Effects of essential amino acid supplementation on exercise and performance. J. Int. Soc. Sports Nutr..

[26] Morton J.P., Hearris M., Fell M.J., Owens D.J., Halson S., Trommelen J. (2025). UCI Sports Nutrition Project: Nutritional Periodization: Strategies to Enhance Training Adaptation and Recovery. Int. J. Sport Nutr. Exerc. Metab..

[27] Stellingwerff T., Morton J.P., Burke L.M. (2019). A Framework for Periodized Nutrition for Athletics. Int. J. Sport. Nutr. Exerc. Metab..

[28] Mănescu D.C. (2010). Alimentaţia în Fitness şi Bodybuilding.

[29] Gejl K.D., Nybo L. (2021). Performance effects of periodized carbohydrate restriction in endurance trained athletes—A systematic review and meta-analysis. J. Int. Soc. Sports Nutr..

[30] Podlogar T., Wallis G.A. (2022). New Horizons in Carbohydrate Research and Application for Endurance Athletes. Sports Med..

[31] Schumann M., Feuerbacher J.F., Sünkeler M., Freitag N., Rønnestad B.R., Doma K., Lundberg T.R. (2022). Compatibility of Concurrent Aerobic and Strength Training for Skeletal Muscle Size and Function: An Updated Systematic Review and Meta-Analysis. Sports Med..

[32] Huiberts R.O., Wüst R.C.I., van der Zwaard S. (2024). Concurrent Strength and Endurance Training: A Systematic Review and Meta-Analysis on the Impact of Sex and Training Status. Sports Med..

[33] Jones T.W., Eddens L., Kupusarevic J., Simoes D.C.M., Furber M.J.W., van Someren K.A., Howatson G. (2021). Aerobic exercise intensity does not affect the anabolic signaling following resistance exercise in endurance athletes. Sci. Rep..

[34] Lee M.J., Caruana N.J., Saner N.J., Kuang J., Stokes T., McLeod J.C., Oikawa S.Y., Bishop D.J., Bartlett J.D., Phillips S.M. (2024). Resistance-only and concurrent exercise induce similar myofibrillar protein synthesis rates and associated molecular responses in moderately active men before and after training. FASEB J..

[35] Denadai B.S., Greco C.C. (2025). Muscle fatigue and interference phenomenon during concurrent aerobic and strength training: An alternative hypothetical model. Med. Hypotheses.

[36] Wolff C.A., Esser K.A. (2019). Exercise Timing and Circadian Rhythms. Curr. Opin. Physiol..

[37] Mansingh S., Handschin C. (2022). Time to Train: The Involvement of the Molecular Clock in Exercise Adaptation of Skeletal Muscle. Front. Physiol..

[38] Aoyama S., Shibata S. (2020). Time-of-Day-Dependent Physiological Responses to Meal and Exercise. Front. Nutr..

[39] Negri M., Pivonello C., Amatrudo F., Cimmino F., Trinchese G., Vetrani C., Iaccarino G., Pivonello R., Mollica M.P., Colao A. (2025). Effects of Chrono-Exercise and Chrono-Nutrition on Muscle Health: Understanding the Molecular Mechanisms Activated by Timed Exercise and Consumption of Proteins and Carbohydrates. Nutr. Rev..

[40] Mishima T., Takenaka Y., Hashimoto-Hachiya A., Tanigawa Y., Suzuki N., Oishi K., Ogasawara R. (2025). Time-of-day effect of high-intensity muscle contraction on mTOR signaling and protein synthesis in mice. Sci. Rep..

[41] Henriquez-Olguin C., Meneses-Valdes R., Jensen T.E. (2020). Compartmentalized muscle redox signals controlling exercise metabolism—Current state, future challenges. Redox Biol..

[42] Zhou Y., Zhang X., Baker J.S., Davison G.W., Yan X. (2024). Redox signaling and skeletal muscle adaptation during aerobic exercise. iScience.

[43] Powers S.K., Radak Z., Ji L.L., Jackson M. (2024). Reactive oxygen species promote endurance exercise-induced adaptations in skeletal muscles. J. Sport Health Sci..

[44] Ji L.L., Yeo D. (2022). Maintenance of NAD^+^ Homeostasis in Skeletal Muscle during Aging and Exercise. Cells.

[45] Lamb D.A., Moore J.H., Mesquita P.H.C., Smith M.A., Vann C.G., Osburn S.C., Fox C.D., Lopez H.L., Ziegenfuss T.N., Huggins K.W. (2020). Resistance training increases muscle NAD^+^ and NADH concentrations as well as NAMPT protein levels and global sirtuin activity in middle-aged, overweight, untrained individuals. Aging.

[46] Wang L., Meng Q., Su C.-H. (2024). From Food Supplements to Functional Foods: Emerging Perspectives on Post-Exercise Recovery Nutrition. Nutrients.

[47] Mănescu D.C. (2016). Nutritional tips for muscular mass hypertrophy. Marathon.

[48] Margolis L.M., Allen J.T., Hatch-McChesney A., Pasiakos S.M. (2021). Coingestion of Carbohydrate and Protein on Muscle Glycogen Synthesis after Exercise: A Meta-analysis. Med. Sci. Sports Exerc..

[49] Trommelen J., van Lieshout G.A., Nyakayiru J., Holwerda A.M., Smeets J.S., Hendriks F.K., van Kranenburg J.M., Zorenc A.H., Senden J.M., Goessens J.P. (2023). The anabolic response to protein ingestion during recovery from exercise has no upper limit in magnitude and duration in vivo in humans. Cell Rep. Med..

[50] Podlogar T., Shad B.J., Seabright A.P., Odell O.J., Lord S.O., Civil R., Salgueiro R.B., Shepherd E.L., Lalor P.F., Elhassan Y.S. (2023). Postexercise muscle glycogen synthesis with glucose, galactose, and combined galactose-glucose ingestion. Am. J. Physiol.—Endocrinol. Metab..

[51] Ross R., Goodpaster B.H., Koch L.G., Sarzynski M.A., Kohrt W.M., Johannsen N.M., Skinner J.S., Castro A., Irving B.A., Noland R.C. (2019). Precision exercise medicine: Understanding exercise response variability. Br. J. Sports Med..

[52] Noone J., Mucinski J.M., DeLany J.P., Sparks L.M., Goodpaster B.H. (2024). Understanding the variation in exercise responses to guide personalized physical activity prescriptions. Cell Metab..

[53] Bonafiglia J.T., Preobrazenski N., Gurd B.J. (2021). A Systematic Review Examining the Approaches Used to Estimate Interindividual Differences in Trainability and Classify Individual Responses to Exercise Training. Front. Physiol..

[54] Hrubeniuk T.J., Bonafiglia J.T., Bouchard D.R., Gurd B.J., Sénéchal M. (2022). Directions for Exercise Treatment Response Heterogeneity and Individual Response Research. Int. J. Sports Med..

[55] Chrzanowski-Smith O.J., Piatrikova E., Betts J.A., Williams S., Gonzalez J.T. (2020). Variability in exercise physiology: Can capturing intra-individual variation help better understand true inter-individual responses?. Eur. J. Sport Sci..

[56] Muñoz Fabra E., Díez J.-L., Bondia J., Laguna Sanz A.J. (2021). A Comprehensive Review of Continuous Glucose Monitoring Accuracy during Exercise Periods. Sensors.

[57] Düking P., Zinner C., Trabelsi K., Reed J.L., Holmberg H.-C., Kunz P., Sperlich B. (2021). Monitoring and adapting endurance training on the basis of heart rate variability monitored by wearable technologies: A systematic review with meta-analysis. J. Sci. Med. Sport.

[58] Mănescu D.C., Mănescu A.M. (2025). Artificial Intelligence in the Selection of Top-Performing Athletes for Team Sports: A Proof-of-Concept Predictive Modeling Study. Appl. Sci..

[59] Xuan X., Chen C., Molinero-Fernandez A., Ekelund E., Cardinale D., Swarén M., Wedholm L., Cuartero M., Crespo G.A. (2023). Fully Integrated Wearable Device for Continuous Sweat Lactate Monitoring in Sports. ACS Sens..

[60] Dolson C.M., Harlow E.R., Phelan D.M., Gabbett T.J., Gaal B., McMellen C., Geletka B.J., Calcei J.G., Voos J.E., Seshadri D.R. (2022). Wearable Sensor Technology to Predict Core Body Temperature: A Systematic Review. Sensors.

[61] Solem K., Clauss M., Jensen J. (2025). Glycogen supercompensation in skeletal muscle after cycling or running followed by a high carbohydrate intake the following days: A systematic review and meta-analysis. Front. Physiol..

[62] Prieto-Bellver G., Diaz-Lara J., Bishop D.J., Fernández-Sáez J., Abián-Vicén J., San-Millan I., Santos-Concejero J. (2024). A Five-Week Periodized Carbohydrate Diet Does Not Improve Maximal Lactate Steady-State Exercise Capacity and Substrate Oxidation in Well-Trained Cyclists compared to a High-Carbohydrate Diet. Nutrients.

[63] Nøst H.L., Aune M.A., van den Tillaar R. (2024). The Effect of Polarized Training Intensity Distribution on Maximal Oxygen Uptake and Work Economy Among Endurance Athletes: A Systematic Review. Sports.

[64] Yin M., Li H., Bai M., Liu H., Chen Z., Deng J., Deng S., Meng C., Vollaard N.B.J., Little J.P. (2024). Is low-volume high-intensity interval training a time-efficient strategy to improve cardiometabolic health and body composition? A meta-analysis. Appl. Physiol. Nutr. Metab..

[65] Bodine S.C., Stitt T.N., Gonzalez M., Kline W.O., Stover G.L., Bauerlein R., Zlotchenko E., Scrimgeour A., Lawrence J.C., Glass D.J. (2001). Akt/mTOR pathway is a crucial regulator of skeletal muscle hypertrophy and can prevent muscle atrophy in vivo. Nat. Cell Biol..

[66] Hearris M.A., Owens D.J., Strauss J.A., Shepherd S.O., Sharples A.P., Morton J.P., Louis J.B. (2020). Graded reductions in pre-exercise glycogen concentration do not augment exercise-induced nuclear AMPK and PGC-1α protein content in human muscle. Exp. Physiol..

[67] Russo I., Della Gatta P.A., Garnham A., Porter J., Burke L.M., Costa R.J.S. (2021). The Effects of an Acute “Train-Low” Nutritional Protocol on Markers of Recovery Optimization in Endurance-Trained Male Athletes. Int. J. Sports Physiol. Perform..

[68] Martin R.A., Viggars M.R., Esser K.A. (2023). Metabolism and exercise: The skeletal muscle clock takes centre stage. Nat. Rev. Endocrinol..

[69] Naderi A., Gobbi N., Ali A., Berjisian E., Hamidvand A., Forbes S.C., Koozehchian M.S., Karayigit R., Saunders B. (2023). Carbohydrates and Endurance Exercise: A Narrative Review of a Food First Approach. Nutrients.

[70] Baetica A.A., Westbrook A.M., El-Samad H. (2019). Control theoretical concepts for synthetic and systems biology. Curr. Opin. Syst. Biol..

[71] Suen T., Navlakha S. (2022). A feedback control principle common to several biological and engineered systems. J. R. Soc. Interface.

[72] Stoica G.-D., Stoian M. (2022). Durability, Circularity and Sustainability in the Food Market—A Bibliometric Analysis. Proc. Int. Conf. Bus. Excell..

[73] Brady S.S., Brubaker L., Fok C.S., Gahagan S., Lewis C.E., Lewis J., Lowder J.L., Nodora J., Stapleton A., Palmer M.H. (2020). Development of Conceptual Models to Guide Public Health Research, Practice, and Policy: Synthesizing Traditional and Contemporary Paradigms. Health Promot. Pract..

[74] Mănescu D.C. (2025). Computational Analysis of Neuromuscular Adaptations to Strength and Plyometric Training: An Integrated Modeling Study. Sports.

[75] (2021). ain low” energy availability strategy on the physiological adaptations to a home-based high-intensity interval training program: A randomized control trial. PLoS ONE.

[76] Mănescu A.M., Mănescu D.C. (2025). Self-Supervised Gait Event Detection from Smartphone IMUs for Human Performance and Sports Medicine. Appl. Sci..

[77] Bowler S.M., Dixon B., Monk T., Highton J., Rowlands D.S. (2023). Use of Continuous Glucose Monitors in Sport: Possible Applications and Considerations. Int. J. Sport Nutr. Exerc. Metab..

[78] Kiviniemi A.M., Hautala A.J., Kinnunen H., Tulppo M.P. (2007). Endurance Training Guided Individually by Daily Heart Rate Variability Measurements. Eur. J. Appl. Physiol..

[79] Mentzoni B. (2024). Accuracy and agreement of 4 handheld blood lactate analyzers. Eur. J. Appl. Physiol..

[80] Tricco A.C., Lillie E., Zarin W., O’Brien K.K., Colquhoun H., Levac D., Moher D., Peters M.D.J., Horsley T., Weeks L. (2018). PRISMA Extension for Scoping Reviews (PRISMA-ScR): Checklist and Explanation. Ann. Intern. Med..

[81] Grames E.M., Schwartz D., Elphick C.S. (2022). A Systematic Method for Hypothesis Synthesis and Conceptual Model Development. Methods Ecol. Evol..

[82] Campbell F., Tricco A.C., Munn Z., Pollock D., Saran A., Sutton A., White H., Khalil H. (2023). Mapping reviews, scoping reviews, and evidence and gap maps (EGMs): The same but different—The “Big Picture” review family. Syst. Rev..

[83] Peters M.D.J., Marnie C., Tricco A.C., Pollock D., Munn Z., Alexander L., McInerney P., Godfrey C.M., Khalil H. (2020). Updated methodological guidance for the conduct of scoping reviews. JBI Evid. Synth..

[84] Wolffe T.A.M., Whaley P., Halsall C., Rooney A.A., Walker V.R. (2019). Systematic evidence maps as a novel tool to support evidence-based decision-making in chemicals policy and risk management. Environ. Int..

[85] Balagué N., Pol R., Torrents C., Ric A., Hristovski R. (2020). Network Physiology of Exercise: Vision and Perspectives. Front. Physiol..

[86] Billman G.E. (2020). Homeostasis: The Underappreciated and Far Too Often Ignored Central Organizing Principle of Physiology. Front. Physiol..

[87] El-Samad H. (2021). Biological feedback control—Respect the loops. Cell Syst..

[88] Sennesh E., Quigley K.S. (2022). Interoception as modeling, allostasis as control. Biol. Psychol..

[89] Inoue A., dos Santos Bunn P., Crivoi do Carmo E., Lattari E., da Silva E.B. (2022). Internal training load perceived by athletes and planned by coaches: A systematic review and meta-analysis. Sports Med.—Open.

[90] Liu H., Yang W., Liu H., Bao D., Cui Y., Ho I.M.K., Li Q. (2023). Criterion-related validity of session rating of perceived exertion scales in athletes: A meta-analysis. BMC Sports Sci. Med. Rehabil..

[91] Uleman J.F., Luijten M., Abdo W.F., Vyrastekova J., Gerhardus A., Runge J., Rod N.H., Verhagen M. (2024). Triangulation for causal loop diagrams: Constructing biopsychosocial models using group model building, literature review, and causal discovery. npj Complex..

[92] Smith J.A.B., Murach K.A., Dyar K.A., Zierath J.R. (2023). Exercise metabolism and adaptation in skeletal muscle. Nat. Rev. Mol. Cell Biol..

[93] Egan B., Sharples A.P. (2023). Molecular responses to acute exercise and their relevance for adaptations in skeletal muscle to exercise training. Physiol. Rev..

[94] Furrer R., Hawley J.A., Handschin C. (2023). The molecular athlete: Exercise physiology from mechanisms to medals. Physiol. Rev..

[95] Barrett L.F., Simmons W.K. (2016). An active inference theory of allostasis and interoception in depression. Philos. Trans. R. Soc. B Biol. Sci..

[96] Impey S.G., Hearris M.A., Hammond K.M., Bartlett J.D., Louis J., Close G.L., Morton J.P. (2018). Fuel for the Work Required: A Theoretical Framework for Carbohydrate Periodization and the Glycogen Threshold Hypothesis. Sports Med..

[97] Marquet L.-A., Brisswalter J., Louis J., Tiollier E., Burke L.M., Hawley J.A., Hausswirth C. (2016). Enhanced endurance performance by periodization of carbohydrate intake: “Sleep low” strategy. Med. Sci. Sports Exerc..

[98] Furrer R., Heim B., Schmid S., Dilbaz S., Adak V., Nordström K.J., Ritz D., Steurer S.A., Walter J., Handschin C. (2023). Molecular control of endurance training adaptation in male mouse skeletal muscle. Nat. Metab..

[99] Furrer R., Handschin C. (2024). Molecular aspects of the exercise response and training adaptation in skeletal muscle. Free Radic. Biol. Med..

[100] Nair V.D., Pincas H., Smith G.R., Zaslavsky E., Ge Y., Amper M.A.S., Vasoya M., Chikina M., Sun Y., Raja A.N. (2024). Molecular adaptations in response to exercise training are associated with tissue-specific transcriptomic and epigenomic signatures. Cell Genom..

[101] Mănescu D.C. (2026). Training Load Oscillation and Epigenetic Plasticity: Molecular Pathways Connecting Energy Metabolism and Athletic Personality. Int. J. Mol. Sci..

[102] Jacques M., Landen S., Sharples A.P., Garnham A., Schittenhelm R., Steele J., Heikkinen A., Sillanpää E., Ollikainen M., Broatch J. (2025). Molecular landscape of sex- and modality-specific exercise adaptation in human skeletal muscle through large-scale multi-omics integration. Cell Rep..

[103] Spaulding H.R., Yan Z. (2022). AMPK and the adaptation to exercise. Annu. Rev. Physiol..

[104] Bobba-Alves N., Juster R.-P., Picard M. (2022). The energetic cost of allostasis and allostatic load. Psychoneuroendocrinology.

[105] Mănescu D.C., Plăstoi C.D., Petre R.L., Mărgărit I.R., Mănescu A.M., Pîrvan A. (2026). Metabolic Overdrive in Elite Sport: A Systems Model of AMPK–mTOR Oscillation, NAD^+^ Economy, and Epigenetic Drift. Int. J. Mol. Sci..

[106] Mehrhof S.Z., Fleming H., Nord C.L. (2025). An interoceptive model of energy allostasis linking metabolic and mental health. Sci. Adv..

[107] Louis J., Marquet L.-A., Tiollier E., Bermon S., Hausswirth C., Brisswalter J. (2016). The impact of sleeping with reduced glycogen stores on immunity and sleep in triathletes. Eur. J. Appl. Physiol..

[108] Mountjoy M., Sundgot-Borgen J.K., Burke L.M., Ackerman K.E., Blauwet C., Constantini N., Lebrun C., Lundy B., Melin A.K., Meyer N.L. (2018). IOC consensus statement on relative energy deficiency in sport (RED-S): 2018 update. Br. J. Sports Med..

[109] Carrasco-Poyatos M., González-Quílez A., Altini M., Granero-Gallegos A. (2022). Heart rate variability-guided training in professional runners: Effects on performance and vagal modulation. Physiol. Behav..

[110] Mănescu A.M., Tudor A.C., Dinciu C.C., Hangu S.Ș., Mărgărit I.R., Tudor V., Mănescu C.O., Ciomag R.V., Rădulescu M.L., Hangu C. (2025). Interpretable Machine Learning on Simulation-Derived Biomechanical Features for Hamstrings–Quadriceps Imbalance Detection in Running. Sports.

[111] Medellín Ruiz J.P., Rubio-Arias J.Á., Clemente-Suarez V.J., Ramos-Campo D.J. (2020). Effectiveness of training prescription guided by heart rate variability versus predefined training for physiological and aerobic performance improvements: A systematic review and meta-analysis. Appl. Sci..

[112] Doyle M.M., Keane M., McGarrigle A., Malone F.D. (2021). A SAS macro for modelling periodic data using cosinor analysis. Comput. Methods Programs Biomed..

[113] Flockhart M., Nilsson L.C.J., Larsen F.J. (2023). Continuous glucose monitoring in endurance athletes: Potential, challenges and research opportunities. Sports Med..

[114] Lundstrom C.J., Foreman N., Baggish A.L. (2023). Practices and applications of heart rate variability monitoring in endurance athletes. Int. J. Sports Med..

[115] Raa I., Sunde G.A., Bolann B., Kvåle R., Eliassen H.S., Wentzel-Larsen T., Heltne J.-K. (2020). Validation of a point-of-care capillary lactate measuring device. Scand. J. Trauma Resusc. Emerg. Med..

[116] Littlejohns L.B., Hill C., Neudorf C. (2021). Diverse approaches to creating and using causal loop diagrams in public health research: Recommendations from a scoping review. Public Health Rev..

[117] Dhirasasna N., Sahin O., Miller R., Hinkel J. (2019). A multi-methodology approach to creating a causal loop diagram. Systems.

[118] Cassidy R., Borghi J., Semwanga A.R., Binyaruka P., Singh N.S., Blanchet K. (2022). How to do (or not to do)… using causal loop diagrams for health system research. Health Policy Plan..

