# Supplementary material for: Training–Fuel Coupling (TFC): A Molecular Sports Nutrition Framework for Energy Availability, Chrono-Nutrition, and Performance Optimization
Source: Nutrients. 2026 Feb 21;18(4):693. doi: 10.3390/nu18040693 (PMC12943547; doi:10.3390/nu18040693)
Supplement: Supplementary file 1 [file nutrients-18-00693-s001.zip › nutrients-4140326-supplementary.pdf]

Supplementary

Conceptual Formalization of the Training–Fuel Coupling Framework

To express the emergent behaviors of the Training–Fuel Coupling (TFC) framework in measurable form, four composite indices were established—each representing a principal regulatory dimension of energetic control. These indices provide standardized, reproducible metrics that can be quantified experimentally or in applied monitoring contexts. Where applicable, variability-, stress-, and feedback-related indices (EVI, ESI, CLPI) were normalized to individual baseline values (z-scores) to ensure within-subject comparability across training cycles, whereas bounded phase-based indices (e.g., AOI) are reported on their natural 0–1 scale [87,88,112].

Energetic Variability Index (EVI) - the EVI quantifies how strongly molecular signaling fluctuates between catabolic (AMPK) and anabolic (mTORC1) dominance across repeated sessions:

$$EVI = \sqrt{\Sigma[(R_{ampk,i+1} - R_{ampk,i}) - (R_{mtor,i+1} - R_{mtor,i})]^2 / (n-1)} \div \sigma_0$$

where $R_{ampk,i}$ and $R_{mtor,i}$ are phosphorylation ratios (phospho/total) for AMPK and mTORC1 in consecutive sessions, and σ_0 is the baseline standard deviation. In practice, EVI can be computed either as (1) the within-microcycle standard deviation of a session-wise dominance metric (e.g., log[RAMPK/RmTOR]), or (2) the standard deviation of session-to-session transitions as formalized above; when reported as a z-score relative to an individual baseline distribution, EVI values may be negative (below-baseline variability).

Interpretation: High EVI (> +0.8 SD) = preserved signaling responsiveness; Low EVI (below baseline; z < 0) = metabolic rigidity.

Energetic Stress Index (ESI) - the ESI integrates adenylate and redox charge into a single descriptor of intracellular metabolic tension:

$$ESI = z\,(AMP / ATP) + z\,(NAD^+ / NADH)$$

where AMP/ATP expresses adenylate energy charge and $NAD^+/NADH$ the redox ratio.

Interpretation: Positive ESI (> +1 SD) = catabolic / AMPK-dominant; Negative ESI (< –1 SD) = anabolic / mTORC1-dominant; Optimal ≈ 0 ± 1 SD.

Adaptive Oscillation Index (AOI) - the AOI quantifies the degree of anti-phase coupling between AMPK and mTORC1 activation across a training microcycle.

Let $\Delta\varphi \in [0, \pi]$ denote the circular phase difference between φAMPK and φmTOR, estimated from cosine (cosinor) fits to their respective phosphorylation time-series.

$AOI = \Delta\varphi / \pi$

Interpretation: AOI ≈ 1 ($\Delta\varphi \approx \pi$) reflects strong anti-phase alignment, indicating optimal alternation between catabolic (AMPK-dominant) and anabolic (mTORC1-dominant) states. AOI ≈ 0 ($\Delta\varphi \approx 0$) indicates in-phase coupling, reflecting loss of metabolic alternation and reduced adaptive efficiency. Indicative thresholds: AOI > 0.7 = coherent anti-phase oscillation; AOI < 0.3 = desynchronization.

Closed-Loop Performance Index (CLPI) - the CLPI expresses the efficiency of feedback regulation linking energetic input, recovery, and performance stability:

$$CLPI = (1 - CV_{session}) \times (\Delta Fuel / \Delta Performance)$$

where $CV_{session}$ is the coefficient of variation of session-quality scores (RPE × [La]), ΔFuel the change in carbohydrate intake or CGM-derived energy availability, and ΔPerformance the relative change in performance output.

Interpretation: CLPI > +0.5 SD = effective self-regulation; CLPI < 0 = unstable feedback or poor recovery integration.

Operational Meaning **-** each index isolates a core layer of control within the TFC framework—variability (EVI), stress polarity (ESI), oscillatory timing (AOI), and feedback efficiency (CLPI). Together they transform the theoretical architecture into a quantifiable system, enabling the systematic testing of hypotheses H1–H4 across molecular, functional, and applied contexts.

All indices are dimensionless; variability-, stress-, and feedback-related indices (EVI, ESI, CLPI) are normalized to individual baseline values (z-scores), whereas bounded phase-based indices (e.g., AOI) are reported on their natural 0–1 scale. Positive composite scores denote efficient adaptive regulation; negative scores indicate desensitization or maladaptive instability.

Operationalization of the Indices

In applied settings, direct molecular quantification of AMPK–mTOR signaling or adenylate ratios is rarely feasible. Therefore, the Training–Fuel Coupling (TFC) framework can be operationalized through validated physiological and technological proxies that approximate the four regulatory dimensions described above [87,88,112–115].

Energetic Variability (EVI) - session-to-session variation in energetic context can be estimated from continuous glucose monitoring (CGM) and heart-rate variability (HRV). The ratio of low- to high-glucose training sessions within a week (Δmean glucose > ±1.5 mmol·L^{-}[illegible]h^{-}[illegible]) and the standard deviation of daily HRV (SDNN or lnRMSSD) provide non-invasive correlates of metabolic diversity. An EVI derived from CGM–HRV variance above +0.8 SD denotes optimal energetic alternation.

Energetic Stress (ESI) - integrates adenylate and redox charge into a single descriptor of intracellular metabolic tension. In applied contexts, it can be inferred from post-exercise lactate, HRV suppression, and CGM glucose drift. High values reflect catabolic (AMPK-dominant) states, negative values anabolic (mTORC1-dominant) recovery; optimal adaptation occurs near equilibrium.

Adaptive Oscillation (AOI) - oscillatory coupling can be inferred from alternating patterns of session type and energetic context. Applying a cosine fit to the 7-day sequence of ESI values yields a phase estimate for metabolic stress and recovery. In applied settings, AOI can be approximated using a phase-based proxy derived from the anti-phase relationship between stress- and recovery-dominant sessions; coherence thresholds (AOIproxy > 0.7) reflect consistent alternation rather than in-phase synchronization.

Closed-Loop Performance (CLPI) - feedback efficiency can be quantified from session-quality variability and fueling adjustments. The parameter uses subjective exertion (RPE), external load (power or

velocity), and fueling data (CGM-derived carbohydrate flux). High CLPI scores reflect adaptive stability—performance maintained with decreasing physiological strain [116–118].

Collectively, these proxy-based computations allow the TFC indices to be estimated using wearable sensors and routine performance data, transforming a mechanistic model into a field-ready monitoring framework. This operationalization enables longitudinal tracking of energetic variability, stress polarity, oscillatory coherence, and feedback efficiency—turning molecular logic into practical decision support for individualized training and nutrition periodization.

Practical estimation of these indices through wearable-derived data is summarized in Table 1, which lists non-invasive biomarkers, computational proxies, and interpretative thresholds for field application of the TFC framework.

Table S1. Practical Biomarkers of Energetic State and TFC Proxy Indices

Index	Primary Biomarker(s)	Device / Method	Operational Formula or Metric	Interpretation	Threshold (indicative)
**Energetic Variability Index (EVI)**	Mean CGM glucose (Δ mmol·L^{-}◎h^{-}◎); HRV (SDNN or lnRMSSD)	Continuous Glucose Monitor (CGM); HRV chest strap or wearable	EVI_{proxy} = SD_z(CGM) + SD_z (HRV)	Captures session-to-session variability in energetic context	> +0.8 SD → optimal adaptive variability; < 0 → metabolic rigidity
**Energetic Stress Index (ESI)**	Blood lactate [La]; HRV suppression; CGM glucose drift	Portable lactate analyzer; HRV sensor; CGM	ESI_{proxy} = z([La]) - z(HRV) - z(ΔGlucose)	Integrates catabolic stress (AMPK) vs. anabolic recovery (mTORC1)	+1 → catabolic (AMPK phase); −1 → anabolic (mTORC1 phase)
**Adaptive Oscillation Index (AOI)**	Sequence of ESI values across 7 days	Computed from daily proxy data	AOIproxy = Δφ / π (Δφ from a cosinor fit to the 7-day ESI proxy time-series)	Measures rhythmic coupling between AMPK and mTORC1 activation	> 0.7 → coherent oscillation; < 0.3 → desynchronization
**Closed-Loop Performance Index (CLPI)**	Session quality (RPE × [La]); fueling adjustment (ΔFuel / ΔPerf)	RPE log; lactate analyzer; CGM or nutrition app	$CLPI_{proxy}$ = (1 – $CV_{session}$) × (ΔFuel / ΔPerf)	Quantifies efficiency of adaptive feedback regulation	> +0.5 SD → stable self-regulation; < 0 → feedback inefficiency
**Composite Energetic Score (optional)**	Weighted mean of standardized indices **(z(EVI)+z(ESI)+ z(AOI)+z(CLPI))/4**	Derived variable	Aggregates system-level adaptive efficiency	High positive → optimal coupling; negative → instability	±1 SD = normal range; > +1 SD = high adaptive coherence

Notes. All indices are dimensionless; EVI, ESI, and CLPI are standardized as within-individual z-scores, whereas AOI is reported on its natural 0–1 scale (phase-based). For the Composite Energetic Score, AOI (or AOIproxy) is additionally standardized (z-scored) prior to aggregation. CGM = continuous glucose monitoring; HRV = heart-rate variability; [La] = post-exercise blood lactate concentration; CV = coefficient of variation; ΔFuel = change in carbohydrate intake or energy availability; ΔPerf = change in session performance output.

Table S2. Validation Landscape for H1 — Energetic Variability and Adaptive Efficiency. Experimental design variables, signaling markers, and validation priorities defining substrate-dependent adaptive modulation.

Dimension	Descriptor	Operational range / marker	Expected molecular bias	Adaptive outcome	Key verification methods
**Energetic context**	Muscle glycogen availability	LOW < 350 mmol·kg^{-1} dw / HIGH > 500 mmol·kg^{-1} dw	LOW → ↑p-AMPK^Thr172, ↑p-ACC^Ser79, ↑PGC-1α; HIGH → ↑p-p70S6K^Thr389, ↑4E-BP1^Thr37/46	Divergent activation of oxidative vs. anabolic signaling pathways	Muscle biopsies 0/1/3 h post-exercise; phospho/total ratios
**Substrate state**	Carbohydrate–protein co-availability	LOW: fasted or depleted; HIGH: CHO 0.5 g·kg^{-1} + EAA 0.3 g·kg^{-1}	Nutrient-rich condition re-activates mTORC1; low substrate sustains AMPK tone	Bias toward recovery (fed) or mitochondrial signaling (depleted)	Controlled pre-exercise feeding; isotopic tracer FSR measurement
**Redox balance**	NAD^+/NADH ratio	1.8–2.8 range	↑NAD^+ activates SIRT1–PGC-1α; ↓NAD^+ favors mTORC1 translation	Fine-tuning of metabolic flexibility	Enzymatic assays (NAD^+/NADH); Western blot for Ac-PGC-1α
**Metabolic readout**	p-AMPK/p-p70S6K ratio	HIGH vs. LOW comparison	Reciprocal activation pattern (AMPK↑ mTORC1↓ or vice versa)	Defines variability of energetic signaling	Densitometric quantification; normalization to total protein
**Computational proxy**	Energetic Variability Index (EVI) = SD of (AMPK/mTORC1 ratio) across sessions	EVI > +0.8 SD → enhanced adaptive responsiveness	Quantifies sensitivity to context variation	Predicts adaptation efficiency	Time-series modeling; intra-individual variance analysis
**Validation priority**	Experimental scope	Acute molecular and short-term training contexts	Tier 1 – acute signaling; Tier 2 – repeated exposure; Tier 3 – cross-modal adaptation	Demonstrates how contextual variation enhances adaptive gain	Crossover HIIT trials; biopsy and performance endpoints
**Potential confounders**	Behavioral and physiological factors	Nutrition timing, fiber type composition, habitual energy intake, recovery status	Alter adaptive bias or dampen variability effect	Controlled feeding, fiber-type matching, standardized recovery window	Repeated-measures design; pre-post balance check

Notes: Glycogen thresholds estimated from train-low literature (250–600 mmol·kg^{-1} dw). Phosphorylation residues per UniProt: AMPK^Thr172, ACC^Ser79, p70S6K^Thr389, 4E-BP1^Thr37/46. NAD^+/NADH ratio serves as a redox proxy for SIRT1–PGC-1α activation. EVI = Energetic Variability Index. Abbreviations: CHO – carbohydrate; EAA – essential amino acids; FSR – fractional synthesis rate; SD – standard deviation.

Validation logic

Prediction - At an equivalent external workload (ΔTSS ≈ 0), metabolic signaling will diverge according to glycogen availability. When glycogen <350 mmol·kg^{-}◎dw, the model predicts ↑p-AMPK^Thr172, ↑p-ACC^Ser79, and ↑PGC-1*α* mRNA, reflecting oxidative remodeling. When >500 mmol·kg^{-}◎dw, the anabolic profile dominates (↑p-p70S6K^Thr389, ↑p-4E-BP1^Thr37/46, ↑protein synthesis).

Mechanistic rationale - Substrate abundance gates molecular control: reduced glycogen raises AMP/ATP and NAD^+/NADH, activating AMPK and SIRT1-PGC-1*α*; sufficient substrate reinstates Rheb–mTORC1 signaling and translational drive. Thus, variability in substrate status explains inter-individual differences in adaptation under identical external training loads.

Experimental test - Randomized crossover HIIT (8 × 3 min @ 90 % VO_2max) under low- vs. high-glycogen conditions; biopsies at 0/1/3 h.

Primary outcomes: p-AMPK^Thr172, p-p70S6K^Thr389, p-ACC^Ser79 (phospho/total). A Condition × Time interaction (↑AMPK, ↓mTORC1 under low glycogen) with $p < 0.05$ would confirm energetic variability; absence of divergence ($\Delta < 0.2$ SD) would falsify it.

Table S3. Validation Landscape for H2 — Threshold Regulation of Signaling Dominance. Experimental framework for quantifying energetic boundaries, signaling polarity, and bistable metabolic transitions.

Dimension	Descriptor	Operational range / marker	Expected molecular bias	Adaptive outcome	Key verification methods
**Energy threshold**	Muscle glycogen concentration	~300 mmol·kg^{-1} dw boundary	Below threshold → ↑p-AMPK^Thr172, ↓p-p70S6K^Thr389, ↓p-mTOR^Ser2448	Transition from anabolic toward oxidative phenotype	Phosphorylation assays; load-matched strength sessions
**Energy charge**	AMP/ATP ratio	>0.03 (LOW) / <0.02 (HIGH)	↑AMP triggers AMPK autophosphorylation and TSC2-mediated mTORC1 inhibition	↑Fat oxidation, ↓Protein synthesis	LC–MS nucleotide profiling; energy charge quantification
**Redox coupling**	NAD^+/NADH ratio	>2.5 (LOW) / <1.8 (HIGH)	↑NAD^+ promotes SIRT1 activation and PGC-1α deacetylation	↑Mitochondrial biogenesis and oxidative remodeling	Western blot (Ac-PGC-1α); enzymatic assays (CS, COX)
**Metabolic readout**	p-ACC^Ser79 / p-mTOR^Ser2448 ratio	LOW/HIGH comparison	↑p-ACC and ↓p-mTOR indicate metabolic gating	Defines activation–inhibition boundary and threshold crossing	Densitometry; phospho/total normalization
**Computational proxy**	Energetic Stress Index (ESI) = z(AMP/ATP) + z(NAD^+/NADH)	ESI > +1.0 SD → AMPK dominance	Continuous estimate of threshold crossing probability	Predicts binary signaling transitions (on/off)	Systems modeling; ROC analysis; machine-learning classification
**Validation priority**	Model testing tier	Human experimental model	Tier 1 – acute molecular; Tier 2 – repeated exposure; Tier 3 – adaptive trend	Confirms bistability and reversibility of the signaling switch	Sequential trials; biomarker reproducibility across conditions
**Potential confounders**	Inter-individual and environmental modifiers	Context-dependent: nutrition timing, circadian phase, sex	May shift apparent threshold or dampen response magnitude	Controls required for feeding state, chronobiology, and energy availability	Crossover design; standardized diet; matched training load

Notes: Energy charge derived from adenylate pool (ATP + ADP + AMP). Redox ratios measured enzymatically (NAD^+/NADH). Glycogen thresholds from train-low literature (250–600 mmol·kg^{-1} dw). Phosphorylation residues per UniProt: AMPK^Thr172, p70S6K^Thr389, mTOR^Ser2448, ACC^Ser79. Abbreviations: CS – citrate synthase; COX – cytochrome c oxidase; SD – standard deviation; ESI – Energetic Stress Index.

Validation logic

Prediction - Crossing the energetic threshold (~300 mmol·kg^{-}◎ dw; AMP/ATP > 0.03, NAD^+/NADH > 2.5) will flip signaling polarity: ↑p-AMPK^Thr172 and ↑p-ACC^Ser79 coincide with ↓p-mTOR^Ser2448 and ↓p-p70S6K^Thr389. The system transitions from anabolic to catabolic dominance once the energy charge drops below this limit.

Mechanistic rationale - Low glycogen elevates AMP and activates the AMPK–TSC2–Raptor axis, which suppresses mTORC1 until substrate replenishment restores anabolic drive. This reversible bistability explains

sharp shifts in adaptation with small nutritional changes—a molecular switch linking energy state to signaling dominance.

Experimental test - Crossover strength protocol (5 × 5 @ 85 % 1 RM) under LOW (~300 mmol·kg^{-}◎ dw) and HIGH (>500 mmol·kg^{-}◎ dw) glycogen. Biopsies 0/1/3 h; outcomes p-AMPK^Thr172, p-p70S6K^Thr389, p-ACC^Ser79. A clear LOW : HIGH inversion (AMPK↑ / mTORC1↓) validates the threshold; trivial Δ < 0.2 SD refutes.

Table S4. Validation Landscape for H3 — Oscillatory Coupling and Periodized Adaptation. Integrated experimental parameters linking metabolic rhythm, training periodization, and composite adaptive gain.

Dimension	Descriptor	Operational range / marker	Expected molecular bias	Adaptive outcome	Key verification methods
**Cycle architecture**	Catabolic–anabolic alternation	~48 h oscillation: endurance (AMPK) → strength (mTORC1)	Alternating ↑p-AMPK^Thr172 and ↑p-p70S6K^Thr389 phases	Integrated oxidative and hypertrophic adaptation	Controlled microcycle scheduling; session timing verification
**Energetic amplitude**	ΔGlycogen between phases	≈ ±200 mmol·kg^{-1} dw	Greater amplitude → stronger signaling oscillation	↑CS +15 %, ↑COX +12 %, ↑CSA +5–8 %	Glycogen assay; enzymatic quantification; muscle imaging
**Nutritional synchronization**	Feeding window and macronutrient timing	Protein early (0–1 h) with delayed CHO (2–3 h) after low-glycogen session	Leucine and insulin signaling reinforce mTORC1 activation post-AMPK phase	Amplified adaptation efficiency (Δ1RM +3–5 %)	Dietary control; post-exercise metabolic profiling
**Metabolic readout**	Oscillation coherence between AMPK/mTORC1 pathways	Phase shift ≈ π (180° out of phase)	Anti-phase coupling between AMPK and mTORC1 markers	Predicts synergistic adaptation	Cosine-fit modeling; phospho-signature time course
**Computational proxy**	Adaptive Oscillation Index (AOI) = Δϕ / π (phase difference from cosinor-fitted AMPK and mTORC1 time-series)	AOI > 0.7 → coherent anti-phase oscillation	Quantifies systemic oscillatory alignment	Predicts global adaptation score	Signal analysis; cross-correlation algorithms
**Validation priority**	Model scalability	Short-term mesocycle studies (6–8 weeks); athlete-level	Tier 2 – longitudinal training; Tier 3 – systems adaptation	Demonstrates emergent stability from oscillatory control	Randomized intervention trials; performance & biopsy endpoints
**Potential confounders**	Recovery duration, sleep, circadian phase, hormonal fluctuations	Inter-individual variation in oscillation amplitude	Desynchronization reduces adaptive coherence	↑Variance of ΔCS or ΔCSA	Standardized recovery timing; circadian alignment; sleep tracking

Notes*:* Oscillatory coupling refers to anti-phase alternation between AMPK- and mTORC1-dominant states within ~48 h microcycles. Δphase ≈ π indicates maximal metabolic opposition. AOI = Adaptive Oscillation Index, a bounded phase-based metric (0–1) derived from Δphase; Δphase ≈ π indicates maximal anti-phase alternation between AMPK- and mTORC1-dominant states. CS – citrate synthase; COX – cytochrome c oxidase; CSA – cross-sectional area; Δ – within-condition change.

Validation logic

Prediction - Alternating low-glycogen endurance (AMPK phase) and fed strength (mTORC1 phase) sessions over ~48 h cycles will enhance

both oxidative and contractile adaptation. Expected outcomes: ↑CS (+15 %), ↑COX (+12 %), ↑CSA (+5–8 %), and ↑1 RM (+3–5 %) relative to isocaloric constant-fuel training.

Mechanistic rationale - Intentional metabolic oscillation maintains regulatory sensitivity: AMPK activation primes mitochondrial signaling, while subsequent nutrient-fed mTORC1 engagement rebuilds protein mass. The alternation prevents desensitization, synchronizing catabolic and anabolic phases for maximal adaptive yield.

Experimental test - 8-week intervention comparing oscillatory TFC microcycles (train-low → lift-fed) with constant fueling. Assess CS, COX, β-HAD activities; muscle CSA (ultrasound); 1 RM; and 40-min TT. A composite adaptive index ΣZ > +0.5 SD vs. control validates oscillatory coupling; loss of dual gains falsifies it.

Table S5. Validation Landscape for H4 — Closed-Loop Regulation and Feedback Optimization. Systems-level framework for testing dynamic feedback, performance stability, and adaptive efficiency.

Dimension	Descriptor	Operational range / marker	Expected molecular bias	Adaptive outcome	Key verification methods
**Feedback inputs**	Composite physiological signals (RPE × [La] × ΔHRV)	r > 0.6 vs. glycogen depletion; CV < 10 %	Reliable internal sensing of energetic status	Dynamic fueling adjustments improve session quality	Continuous monitoring of RPE, HRV, lactate
**Control algorithm**	Adaptive fueling via CGM-HRV-RPE integration	Δglucose ±1.5 $mmol \cdot L^{-1} \cdot h^{-1}$, HRV LF/HF ratio normalization	Closed-loop modulation of substrate intake → AMPK-mTORC1 balance	Stable performance output across microcycles	Algorithmic feedback loop; wearable integration
**Energetic feedback gain**	Sensitivity of fueling adjustment to physiological drift	Gain coefficient k_feedback ≈ 0.4–0.6	Higher gain → faster correction of energy imbalance	↓Session variability; ↑adaptive efficiency	Regression analysis; signal-response modeling
**Metabolic readout**	p-AMPK^Thr172 amplitude across cycles	↓Amplitude (−20 %) with maintained output	Reduced stress oscillation → adaptive homeostasis	Efficient substrate use with less molecular noise	Serial biopsies; longitudinal phospho-profiling
**Computational proxy**	Closed-Loop Performance Index (CLPI) = stability × gain	CLPI > +0.5 SD → optimized adaptation	Quantifies efficiency of feedback learning	Predicts performance retention across cycles	Time-series analysis; machine-learning prediction
**Validation priority**	Systems-level training studies	Athlete or advanced trainee cohorts; 6–8-week duration	Tier 2 – longitudinal performance; Tier 3 – real-time sensor adaptation	Demonstrates emergent self-regulation	Controlled trials with integrated wearables
**Potential confounders**	Sensor delay, data noise, motivation, hydration status	Context-dependent; may distort feedback accuracy	Delayed or false feedback → suboptimal control	Blunted adaptation or instability	Signal filtering; algorithm calibration; controlled hydration

Notes: *Closed-loop regulation denotes adaptive adjustment of substrate intake and training load based on real-time feedback. Composite index = RPE × [La] × ΔHRV. CGM – continuous glucose monitoring; HRV – heart-rate variability; LF/HF – low/high-frequency spectral ratio. CLPI = Closed-Loop Performance Index, combining feedback gain and stability metrics. CV – coefficient of variation; SD – standard deviation.*

Validation logic

Prediction - If feedback markers (RPE×[La], ΔHRV, CGM Δglucose ± 1.5 mmol·L^{-}◎h^{-}◎) reflect energetic state, integrating them into a closed-loop fueling algorithm should stabilize training quality (↓CV session quality) and accelerate recovery (↓ΔCK 48 h).

Mechanistic rationale - Physiological feedback creates a self-tuning loop: metabolic stress sensed via HRV and CGM modifies substrate intake, shifting the system toward its optimal energy zone. Over repeated cycles, this feedback reduces signal amplitude but improves efficiency—a hallmark of adaptive homeostasis.

Experimental test - 6-week comparative trial: closed-loop (adaptive fueling guided by CGM + HRV + RPE) vs. fixed schedule. Metrics: Δ1 RM, ΔTT, variability of session quality, recovery kinetics. Improvements of +3–5 % strength, +2–3 % endurance, and lower CV ($p < 0.05$) confirm the feedback hypothesis; null AUC ≈ 0.5 falsifies it.

Table S6. Experimental Validation Model for the Training–Fuel Coupling (TFC) Framework

H#	Core construct	Prediction (summary)	Mechanistic rationale	Experimental test (design)	Primary outcomes / falsification criteria
**H1**	Energetic variability → adaptive efficiency	At same external load, signaling diverges with glycogen state: <350 mmol·kg^{-}◎dw → ↑p-AMPK^Thr172 ↑p-ACC^Ser79 ↑PGC-1α; >500 mmol·kg^{-}◎dw → ↑p-p70S6K^Thr389 ↑4E-BP1^Thr37/46.	Substrate state modulates AMP/ATP and NAD⁺/NADH ratios, shifting control between AMPK–SIRT1–PGC-1α and Rheb–mTORC1 pathways.	Randomized crossover HIIT (8×3 min @ 90% VO_2max) under LOW vs HIGH glycogen; biopsies 0/1/3 h.	Condition × Time effect (↑AMPK, ↓mTORC1 in LOW, $p < 0.05$); Δ < 0.2 SD = falsified.
**H2**	Threshold regulation of signaling dominance	Crossing energetic threshold (~300 mmol·kg^{-}◎ dw; AMP/ATP > 0.03, NAD⁺/NADH > 2.5) flips signaling polarity (↑AMPK / ↓mTORC1).	Glycogen depletion activates AMPK–TSC2–Raptor cascade; substrate repletion re-engages mTORC1. System behaves as bistable molecular switch.	Strength crossover (5×5 @ 85% 1RM) under LOW vs HIGH glycogen; biopsies 0/1/3 h.	LOW: AMPK↑, mTORC1↓; HIGH: inverse. Trivial Δ < 0.2 SD = falsified.
**H3**	Oscillatory coupling → dual adaptation	Alternating AMPK-dominant endurance and mTORC1-dominant strength (≈48 h cycle) yields dual enhancement (↑CS +15 %, ↑COX +12 %, ↑CSA +5–8 %).	Periodic metabolic oscillation prevents desensitization, aligning catabolic/anabolic phases to maximize net adaptation.	8-week intervention: oscillatory (TFC microcycle train-low→lift-fed) vs constant-fuel control.	ΣZ > +0.5 SD = validated; loss of dual gains = falsified.
**H4**	Closed-loop regulation → feedback optimization	Feedback indices (RPE×[La], ΔHRV, Δglucose ± 1.5 mmol L^{-}◎ h^{-}◎) predict energetic state ($r > 0.6$) and enable adaptive fueling that stabilizes performance.	Iterative sensing of metabolic stress drives adaptive homeostasis (↓signal amplitude, ↑efficiency).	6-week closed-loop (CGM + HRV + RPE) vs fixed schedule; monitor Δ1RM, ΔTT, session variability.	↑Δ1RM (+3–5 %), ↑ΔTT (+2–3 %), ↓CV session quality ($p < 0.05$) = validated; AUC ≈ 0.5 = falsified.

Notes: AMPK – AMP-activated protein kinase; mTORC1 – mechanistic target of rapamycin complex 1; p- denotes phosphorylated residue; LOW/HIGH – glycogen condition (~300 vs >500 mmol·kg^{-}◎dw).

Table S7. Training–Fuel Coupling (TFC) Decision Matrix — Linking Training Goals, Energetic Context, and Adaptive Outcomes

Training Goal	Session Type	Energetic Context (Glycogen / Redox)	Feeding Strategy	Dominant Signaling Pathway	Expected Adaptive Outcome	Monitoring / Control Variable	Safety / Practical Notes
**Aerobic capacity / mitochondrial biogenesis**	Prolonged endurance (HIIT, tempo runs, long intervals)	Low glycogen (<350 mmol·kg^{-1} dw); NAD⁺/NADH > 2.5; AMP/ATP > 0.03	*Train-low: fasted or CHO-depleted; protein early (0–1 h), CHO delayed (2–3 h)*	↑AMPK / ↑SIRT1–PGC-1α	↑CS, ↑COX, ↑β-HAD; ↑oxidative efficiency	HRV ↓5–10 %, CGM Δglucose < −1 mmol·L^{-1}h^{-1}	Avoid chronic depletion; monitor LEA/RED-S risk
**Strength / hypertrophy**	Resistance or mixed-power sessions (5×5, 8–12RM)	High glycogen (>500 mmol·kg^{-1} dw); NAD⁺/NADH < 1.8	*Lift-fed*: CHO 0.5–1.0 g·kg^{-1} + EAA 0.3 g·kg^{-1} pre/post	↑mTORC1 / ↑p70S6K / ↓AMPK	↑CSA, ↑1RM, ↑protein synthesis	ΔHRV recovery +5–8 %, RPE ≤7/10	Match caloric intake to workload; avoid overfeeding days off
**Concurrent / hybrid training**	Endurance + resistance within 24–48 h	Alternating low → high glycogen microcycle	*Train-low, lift-fed* sequencing	Anti-phase AMPK–mTORC1 oscillation	Dual adaptation (↑oxidative + ↑strength)	AOI > 0.7 (phase coherence)	Maintain 24–48 h separation between opposing sessions (≈48 h when both sessions are high-intensity or recovery markers are unfavorable).
**Metabolic flexibility / body composition**	Mixed metabolic circuit, intervals + moderate-load resistance	Moderate glycogen (~400 mmol·kg^{-1} dw)	*Isoenergetic cycling*: moderate CHO, high protein	Balanced AMPK-mTOR equilibrium	↑Fat oxidation, ↑efficiency, ↓mass gain	ESI ≈ 0, EVI moderate	Ensure micronutrient sufficiency
**Recovery / adaptive rebound**	Active recovery, mobility, rest days	Nutrient-rich, low energy stress	*Recover-high*: CHO 1.0 g·kg^{-1} + EAA 0.4 g·kg^{-1}	↑mTORC1 / ↓AMPK	↑Protein synthesis, glycogen resynthesis	CLPI > +0.5 SD	Avoid excess fatigue or underfeeding
**Monitoring-based adjustment**	Ongoing (wearable data)	Context-dependent	CGM + HRV + RPE adaptive loop	Balanced oscillation (feedback optimization)	↑Adaptive stability, ↓performance variability	Δglucose ±1.5 mmol·L^{-1}h^{-1}; HRV normalized	Calibrate algorithm weekly; sensor lag compensation

Notes: AMPK – AMP-activated protein kinase; mTORC1 – mechanistic target of rapamycin complex 1; NAD⁺/NADH – redox ratio; HRV – heart rate variability; CGM – continuous glucose monitoring; RPE – rating of perceived exertion; AOI – Adaptive Oscillation Index; ESI – Energetic Stress Index; EVI – Energetic Variability Index; CLPI – Closed-Loop Performance Index; CSA – cross-sectional area; CS – citrate synthase; COX –

cytochrome c oxidase; β-HAD – β-hydroxyacyl-CoA dehydrogenase; LEA – low energy availability; RED-S – relative energy deficiency in sport.

Table S8. Exemplary Microcycles Translating the Training–Fuel Coupling (TFC) Framework into Practice

Discipline	Microcycle Structure (5–7 days)	Energetic Logic / Feeding Pattern	Expected Adaptive Focus
**Endurance (Marathon / Triathlon)**	Day 1: Long aerobic (train-low) ǀ Day 2: Rest or technique (recover-high) ǀ Day 3: HIIT (train-moderate) ǀ Day 4: Recovery (feed-high) ǀ Day 5: Tempo or progressive long run (train-low) ǀ Day 6–7: Refuel & taper	Alternating low–high glycogen states (Δ ≈ 200 mmol·kg^{-}◎dw). Post–low sessions: delayed CHO (2–3 h), protein early. Post–high sessions: immediate CHO + EAA.	↑Mitochondrial biogenesis, ↑oxidative efficiency, ↑metabolic flexibility
**Team Sports (Football / Basketball)**	Day 1: Tactical + small-sided games (train-moderate) ǀ Day 2: Speed–power (lift-fed) ǀ Day 3: Aerobic technical (train-low) ǀ Day 4: Rest / active recovery ǀ Day 5: Match-simulation (feed-high) ǀ Day 6–7: Regeneration	Micro-oscillation of glycogen: technical days ≈350 mmol·kg^{-}◎, match days >500 mmol·kg^{-}◎. Use sleep-low 1×/week. Maintain protein 1.6–1.8 g·kg^{-}◎day^{-}◎.	↑Game endurance, ↑recovery kinetics, stable anabolic–oxidative balance
**Strength / Power (Weightlifting / CrossFit)**	Day 1: Fasted mobility + AMPK activation (short aerobic) ǀ Day 2: Strength session (lift-fed) ǀ Day 3: Rest or low-intensity conditioning ǀ Day 4: Power + accessory lifts (feed-high) ǀ Day 5: Low-glycogen hypertrophy (train-low) ǀ Day 6: Full refuel + recovery ǀ Day 7: Optional deload	Controlled alternation of AMPK–mTOR phases within week. High-CHO days align with strength sessions; low-CHO days with metabolic conditioning.	↑Hypertrophy efficiency, ↑substrate turnover, ↓training fatigue

***Notes*:** CHO – carbohydrate; EAA – essential amino acids; HRV – heart rate variability; Δ – within-week glycogen difference. Glycogen ranges based on train-low literature (250–600 mmol·kg^{-}◎dw). Amplitudes should be adjusted for sex, season, and training phase.
